# Heparin-Binding Proteins in the Nanoparticle Corona Enhance Cellular Uptake through Glycocalyx Interactions

**DOI:** 10.1021/acsnano.5c19714

**Published:** 2025-12-16

**Authors:** Paulo H. Olivieri, Jackelinne Y. Hayashi, Ricardo J.S. Torquato, André F. Lima, Thayza P. Pereira, Ismael F. Lima, Fernando L.A. Fonseca, Leo K. Iwai, Helena B. Nader, Alexandre K. Tashima, Giselle Z. Justo, Alioscka A. Sousa

**Affiliations:** † Department of Biochemistry, 28105 Federal University of São Paulo (UNIFESP), São Paulo, São Paulo 04044-020, Brazil; ‡ Laboratory of Applied Toxinology, Center of Toxins, Immune-Response and Cell Signaling LETA/CeTICS, Butantan Institute, São Paulo, São Paulo 05503-900, Brazil; § Pharmaceutical Sciences Department, Federal University of São Paulo (UNIFESP), Diadema, São Paulo 09913-030, Brazil; ∥ Clinical Laboratory Department, Faculty of Medicine of ABC (FMABC), Santo Andre, São Paulo 09060-650, Brazil

**Keywords:** nanoparticles, protein corona, heparan sulfate, glycocalyx, cell uptake

## Abstract

Nanoparticles (NPs) designed for intracellular delivery must first navigate the cell-surface glycocalyx before reaching the plasma membrane for internalization. Here, we hypothesized that the glycocalyx can both hinder NP uptake via a barrier effect and enhance uptake by providing recognition sites for corona proteins. To dissect these opposing mechanisms, we prepared NPs with plasma protein coronas either enriched or depleted in heparin-binding proteins (HBPs), along with model coronas containing selected HBPs or non-HBPs. Biophysical assays confirmed strong heparin interactions for HBP-rich NPs, whereas HBP-poor NPs showed weak or no binding. To assess the role of corona–glycocalyx interactions in NP uptake, we used glycocalyx-depleted cells, a chemical inhibitor, and heparin and antithrombin competition assays. We found that canonical HBPs within the protein corona, including antithrombin, apolipoprotein E, and platelet factor 4, significantly enhanced NP surface retention and internalization through protein–glycocalyx interactions. In contrast, HBP-poor NPs showed weak or no interactions with the glycocalyx and, correspondingly, reduced uptake. Significantly, these findings also extended to physiologically derived coronas from control and dyslipidemic sera, with the latter producing HBP-enriched coronas that bound more strongly to heparin and promoted more efficient glycocalyx-dependent NP uptake. These findings highlight the underappreciated role of the glycocalyx in actively engaging with coronal HBPs to drive efficient NP uptake. This insight underscores the need to expand corona engineering beyond membrane receptor interactions, incorporating strategies that optimize glycocalyx interactions for more effective NP delivery.

## Graphical abstract

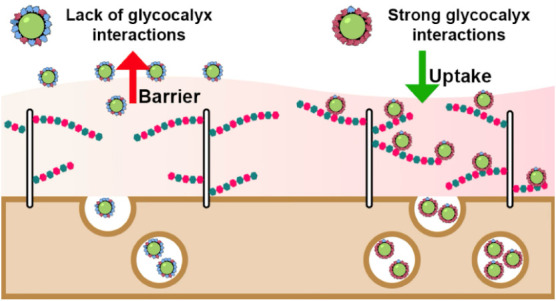

Nanoparticles (NPs) are recognized for their wide-ranging biomedical applications, such as targeted drug and gene delivery. However, the behavior and effectiveness of NPs in biological systems are significantly influenced by the formation of a biomolecular corona upon their exposure to biological fluids.^1–3^ Composed mainly of adsorbed proteins, this corona gives the NPs a biological identity and directly impacts their recognition and internalization by cells.^4–7^ This understanding has given rise to a long-standing paradigm in nanomedicine, whereby NP uptake can be regulated by engineering corona-mediated interactions with cell-surface receptors.^8,9^

However, NP recognition and uptake are complex processes influenced by many factors. One often-overlooked aspect is that NPs must first navigate the cellular glycocalyx before reaching the plasma membrane and undergoing internalization.^10–13^ The glycocalyx is a dense and negatively charged layer of glycoconjugates that coats the cell surface.^10,11^ It is composed of glycoproteins, glycolipids and proteoglycans, the latter consisting of membrane-associated core proteins covalently linked to glycosaminoglycans (GAGs) such as heparan sulfate (HS) and chondroitin sulfate (CS). HS proteoglycans (HSPGs) are a major component of the glycocalyx, given their abundance and chemical diversity. In addition, HSPGs function as receptors or coreceptors for a wide variety of HS-binding proteins (HSBPs), spanning a broad range of binding affinities and specificities.^14–18^

Although universally present on cell surfaces, the glycocalyx has only recently gained attention for its role in NP uptake.^19–27^ It can be theorized that the glycocalyx may serve as a molecular sieve, restricting NP access to the cell surface through steric hindrance and/or electrostatic repulsion.^19,21,22,24,25^ On the other hand, the glycocalyx may provide recognition sites for NP surfaces and corona proteins, thereby facilitating NP entrapment, accumulation at the cell surface, and subsequent uptake.^20,23,28–31^ This duality underscores the need for further exploration into the glycocalyx’s multifaceted role in NP–cell interactions.

Importantly, HSBPs are commonly found within the protein corona of NPs. A clinically relevant example is the adsorption of apolipoprotein E (APOE) onto the surface of Patisiran (Onpattro), a lipid NP formulation carrying therapeutic short interfering RNA.^32^ The adsorbed APOE binds to lipoprotein receptors on hepatocytes, triggering NP internalization via endocytosis. However, the extent to which APOE or other HSBPs may regulate NP uptake through direct binding to cell-surface GAGs remains mostly unexplored. In that regard, we note that APOE binding to HSPGs constitutes a key step in the efficient hepatic uptake of lipoprotein remnants,^33,34^ which suggests a potential role for APOE in glycocalyx-mediated NP uptake as well.^35^

Given this background, we set out to elucidate the role of heparin-binding proteins (HBPs) in NP uptake, noting that heparin is a highly sulfated GAG commonly used as a surrogate for HS. We established a model system using 50 nm silica NPs coated with various types of HBP-rich and HBP-poor protein coronas. Silica NPs were chosen for their clinical potential and their widespread use in protein corona and NP uptake studies.^36^ NP uptake was assessed under various conditions, including cells with intact or glycocalyx-deficient surfaces and cells treated with exogenous agents to modulate corona–glycocalyx interactions. Collectively, our findings reveal a critical role for coronal HBPs in promoting efficient NP uptake via glycocalyx interactions.

## Results and Discussion

### Human Plasma Fractionation

We first prepared the biofluids required to form unique coronas either enriched or depleted of HBPs (Figure 1A). This was achieved by fractionating human plasma using heparin affinity chromatography. Plasma was first dialyzed against an equilibration buffer containing 250 mM NaCl to minimize weak electrostatic interactions with the heparin matrix. The dialyzed plasma was labeled as “CTR”. During chromatography, HBPs were selectively retained on the column and subsequently eluted using 2 M NaCl. The eluted fraction was referred to as “HBP+”, while the unbound plasma, depleted of HBPs, was designated “HBP–” (Figure 1B). The distinct biofluids (HBP+, HBP–, and CTR) were desalted and maintained in sodium phosphate buffer with 150 mM NaCl.

**Figure 1:**
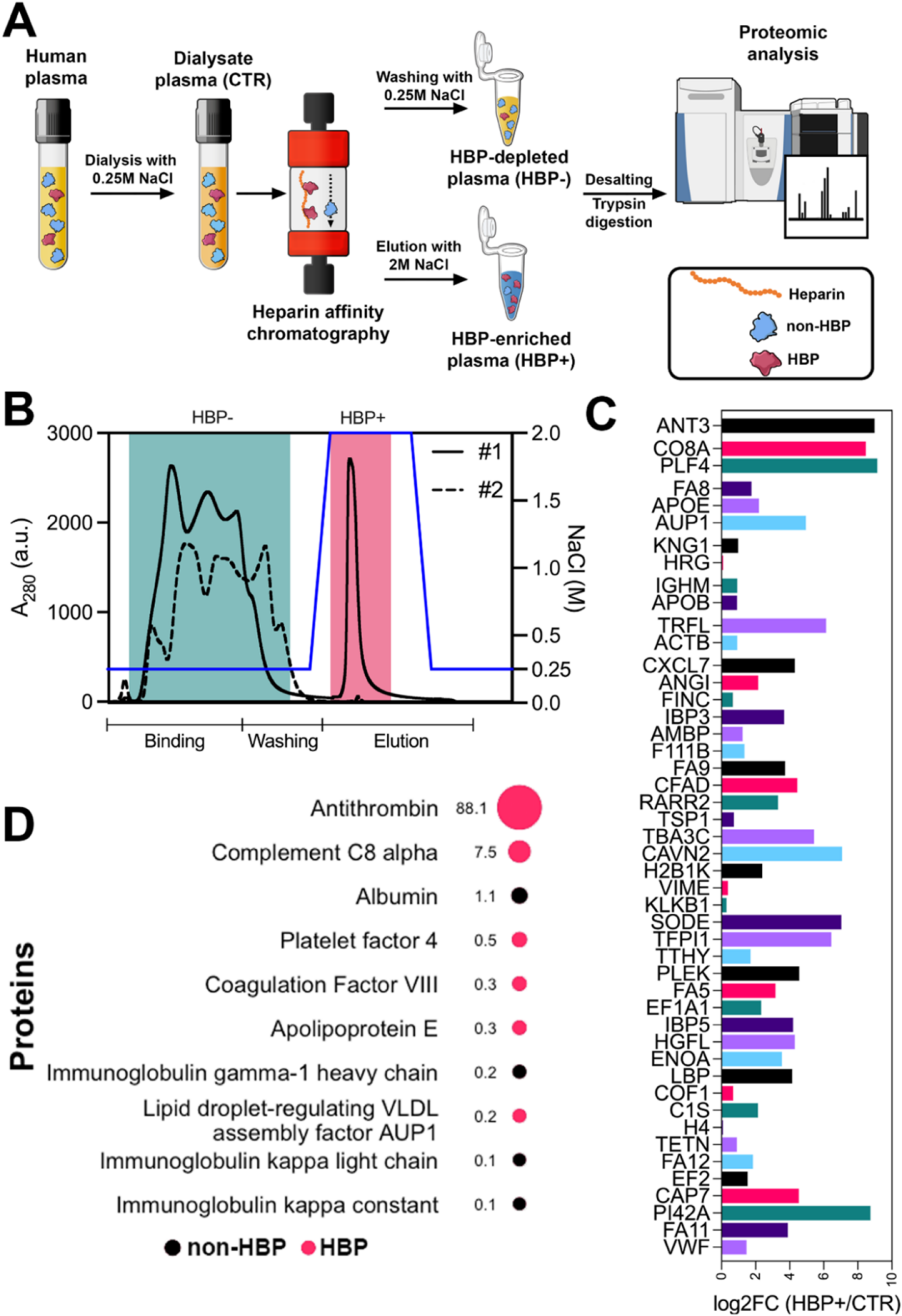
Preparation and mass spectrometry characterization of plasma-derived biofluids used for protein corona formation. (A) Overview of the heparin affinity chromatography workflow used to generate biofluids either enriched (HBP+), depleted (HBP−), or containing unaltered levels (CTR) of HBPs. The resulting biofluids were analyzed by mass spectrometry. (B) Chromatographic elution profile illustrating the isolation of the HBP+ fraction from plasma (solid line #1). Reloading the HBP– fraction yielded no corresponding peak (dashed line #2), confirming depletion of HBPs. (C) Mass spectrometry identification of HBPs in the HBP+ biofluid, based on enrichment relative to the CTR biofluid. (D) Top 10 proteins identified in the HBP+ biofluid, along with their respective RPA values. Some illustrations in panel (A) were adapted from the NIAID NIH BioArt collection (bioart.niaid.nih.gov/bioart).^37^.

The biofluids were then analyzed by mass spectrometry, and a complete list of identified proteins is provided in Suppl. Table S1. As shown in Figure 1C, 47 out of 172 proteins were enriched in the HBP+ biofluid relative to the CTR biofluid, according to the criterion log2 fold change (log2FC) > 0. This subset included several well-known HBPs such as antithrombin (AT), APOE, platelet factor 4 (PF4), and lactoferrin. However, some proteins known to interact with heparin were not among the enriched group. This likely reflects our experimental conditions, particularly the use of 250 mM NaCl, which disrupted weak electrostatic interactions between heparin and potential protein binders. Figure 1D depicts the top 10 proteins identified in the HBP+ biofluid, along with their respective relative protein abundance (RPA) values. AT was the most abundant HBP (88.1% of the total RPA), followed by complement protein C8. Other known HBPs, such as PF4 and APOE, were detected at lower levels. As expected, albumin and immunoglobulins—abundant plasma proteins not typically classified as HBPs—were also present in the HBP+ biofluid in low amounts.

### Protein Corona Formation and Characterization

Silica NPs (50 nm) were incubated with HBP+, HBP–, or CTR biofluids to form distinct protein coronas, resulting in NP_HBP+, NP_HBP–, and NP_CTR (Figure 2A). Corona-coated NPs were characterized for hydrodynamic diameter and surface charge using dynamic light scattering (DLS) and zeta potential (ZP) measurements (Figure 2B-C and Suppl. Table S2). DLS revealed an increase in NP size after corona formation, while ZP measurements showed that coated NPs were less negatively charged than bare NPs. Protein adsorption was further confirmed using the micro-BCA assay (Suppl. Table S2). Taken together, these results validated successful protein corona formation around the silica NPs.

**Figure 2:**
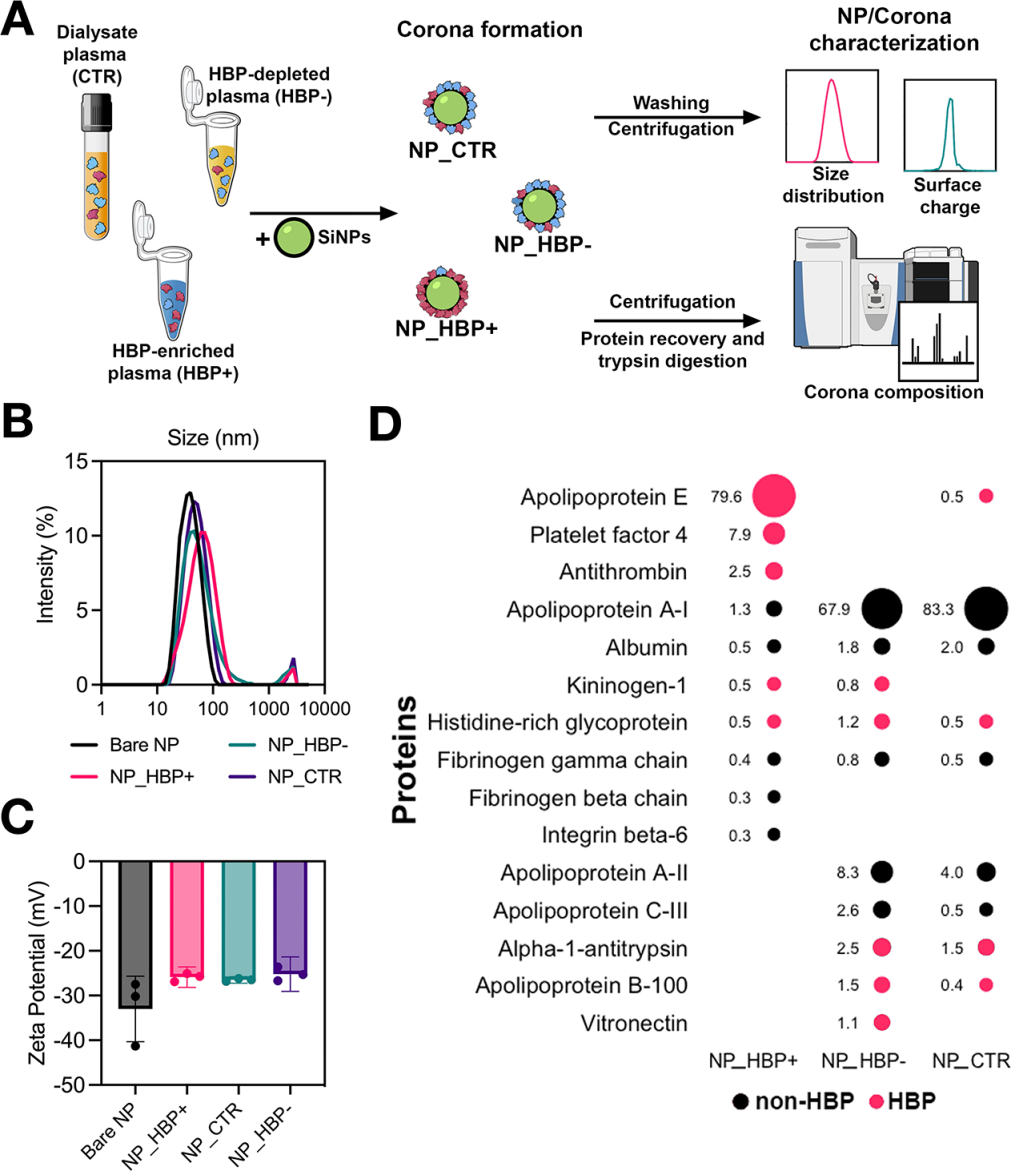
NP characterization and proteomic analysis of the protein corona. (A) Schematic overview of the preparation and characterization of NP_HBP+, NP_HBP–, and NP_CTR. (B) Hydrodynamic diameter distributions of the NPs determined by DLS. (C) Surface charge measurements of the NPs determined by ZP analysis. (D) Top 10 corona proteins identified by mass spectrometry for each NP type, with RPA values indicated. Proteins reported in the literature as HBPs are highlighted in red. Some illustrations in panel (A) were adapted from the NIAID NIH BioArt collection (bioart.niaid.nih.gov/bioart).^37^.

Next, we characterized the corona compositions by mass spectrometry (Figure 2A). A total of 369 distinct proteins were identified, with the most abundant listed in Suppl. Table S3. Figure 2D ranks the top 10 proteins in each corona and their respective RPA values, with HBPs highlighted in red. While the three coronas shared many proteins, key differences also emerged. Apolipoprotein A1 (APOA1) was highly enriched in NP_CTR and NP_HBP–, but constituted only a minor fraction in NP_HBP+. In contrast, APOE was markedly enriched in NP_HBP+ but represented only a minor fraction in NP_CTR and NP_HBP–. APOE is a key component of lipoprotein remnants that binds to low-density lipoprotein (LDL) receptors and mediates their high-capacity receptor-mediated uptake.^38,39^ APOA1, in turn, is a key component of HDL involved in reverse cholesterol transport to hepatocytes through interactions with scavenger receptor type B1, but without requiring HDL endocytosis.^40,41^ Notably, the RPA of APOA1 in NP_CTR and APOE in NP_HBP+ reached nearly 80%. We further note that in NP_HBP+, APOE, PF4, and AT together accounted for 90% of the total RPA. In contrast, HBPs identified among the top 10 proteins in NP_CTR and NP_HBP– contributed <10% of the total RPA.

### Heparin Interactions with Corona-Coated NPs

Having confirmed the formation of HBP-rich and HBP-poor corona-coated NPs, we next investigated their interactions with heparin. To this end, we implemented a simple DLS-based method to assess NP–heparin interactions. The underlying hypothesis was that heparin binding to the protein corona would bridge NPs and promote aggregation, whereas lack of binding would result in little to no aggregation (Figure 3A). As shown in Figure 3B, both NP_HBP+ and NP_CTR exhibited pronounced aggregation in the presence of heparin concentrations above 1 μM. The heparin-mediated aggregation of NP_CTR was surprising, given its low HBP content. At 10 nM heparin, however, NP_CTR aggregated less than NP_HBP+. In contrast to these findings, NP_HBP– showed no aggregation in the presence of heparin (Figure 3B), despite only modest differences in corona composition compared to NP_CTR.

**Figure 3:**
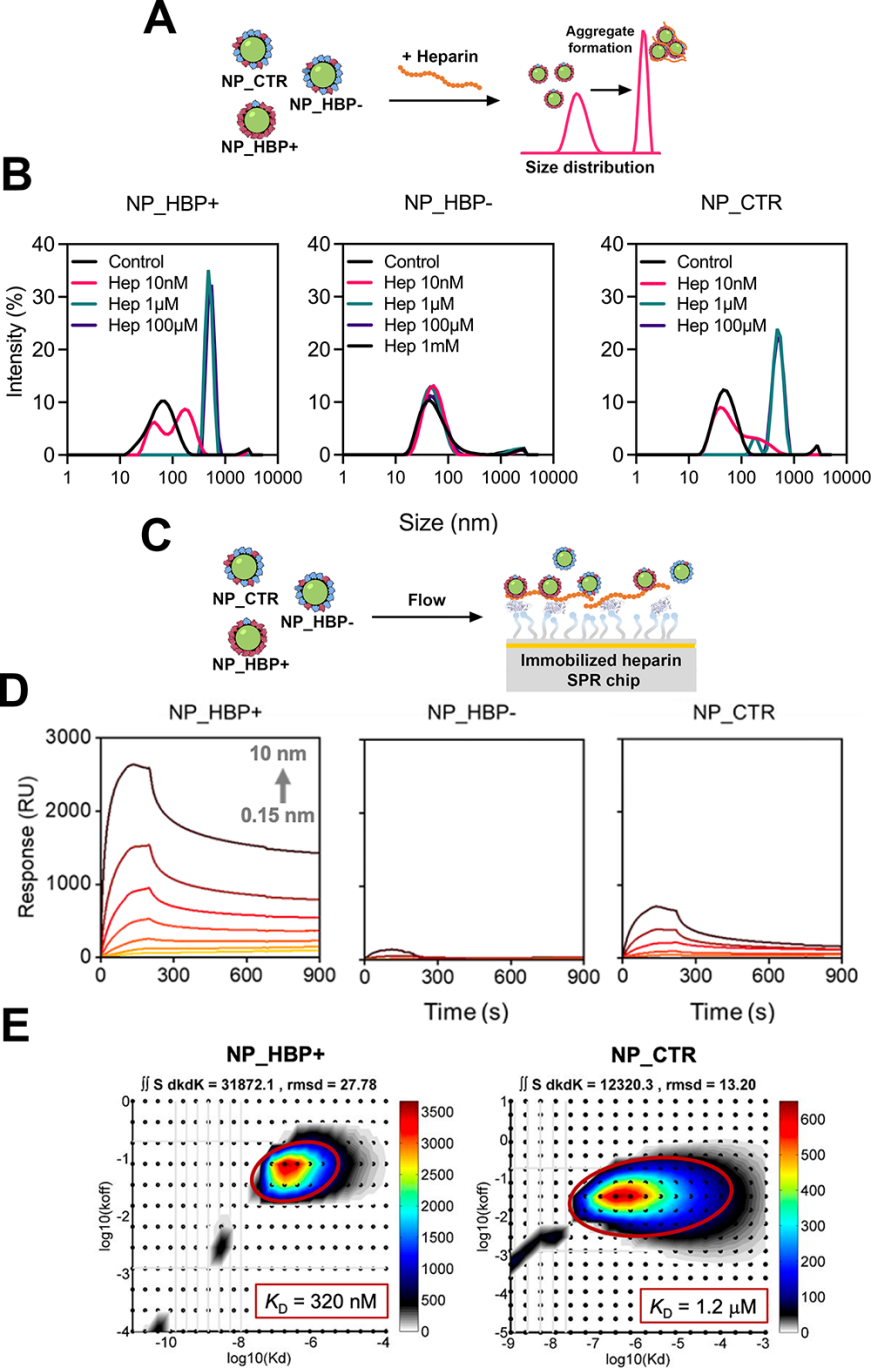
Characterization of NP–heparin interactions. (A) Overview of the DLS-based method to assess heparin-mediated NP aggregation. (B) DLS results after incubation of NPs (250 μg mL^–1^) with increasing concentrations of heparin. Heparin binding to NP_HBP+ and NP_CTR induced NP bridging and aggregation, whereas lack of binding to NP_HBP– prevented their aggregation. (C) SPR setup for analysis of NP–heparin interactions. Biotinylated heparin was immobilized on a streptavidin-coated sensor chip, and NPs were injected over the surface. (D) SPR binding traces showing differential binding responses for NP_HBP+, NP_HBP–, and NP_CTR. (E) Quantitative analysis of NP_HBP+ and NP_CTR binding to heparin using a continuous surface-site distribution model. Shown are the corresponding affinity and dissociation rate constant distributions. In each case, a single binding site is identified (red oval), and the extracted *K*_D_ value is indicated. Some illustrations in panels (A) and (C) were adapted from the NIAID NIH BioArt collection (bioart.niaid.nih.gov/bioart).^37^.

We next employed surface plasmon resonance (SPR) to gain more quantitative insights into NP–heparin interactions (Figure 3C). Heparin was immobilized on the sensor chip, and the NPs were injected over the surface. The binding traces for NP_HBP+ revealed the highest signal responses, followed by NP_CTR, whereas NP_HBP– yielded only weak signals (Figure 3D). These semiquantitative differences imply varying heparin-binding affinities across the NP formulations. For both NP_HBP+ and NP_CTR (though with larger uncertainty for the latter), we were able to fit the data and estimate the apparent binding affinities (*K*_D_), obtaining values of approximately 320 nM for NP_HBP+ and 1.2 μM for NP_CTR (Figure 3E and Suppl. Figure S1).

To conclude this part, we successfully generated an HBP-rich corona-coated NP (NP_HBP+) with high affinity for heparin, along with an HBP-poor counterpart (NP_HBP−) with no detectable heparin binding. We also produced an HBP-poor NP (NP_CTR) that nonetheless retained some capacity to interact with heparin. These differences can be attributed solely to corona composition, as all three NPs had comparable sizes and similar values of ZP.

### NP Surface Adhesion and Uptake

We next investigated how the HBP content and heparin-binding properties of our tailored NPs affected their cellular association and uptake (Figure 4A). Chinese Hamster Ovary (CHO) cells were used as a model system because of their established role in studying GAG-mediated internalization.^42–45^ Both wild-type CHO-K1 and the GAG-deficient mutant pgsA-745 were included.^45^ Cells were incubated with NP_HBP+, NP_HBP–, or NP_CTR, and uptake was quantified via flow cytometry. Experiments were conducted in serum-free media to isolate the impact of corona composition on uptake and avoid confounding effects from excess free proteins.^46^

**Figure 4:**
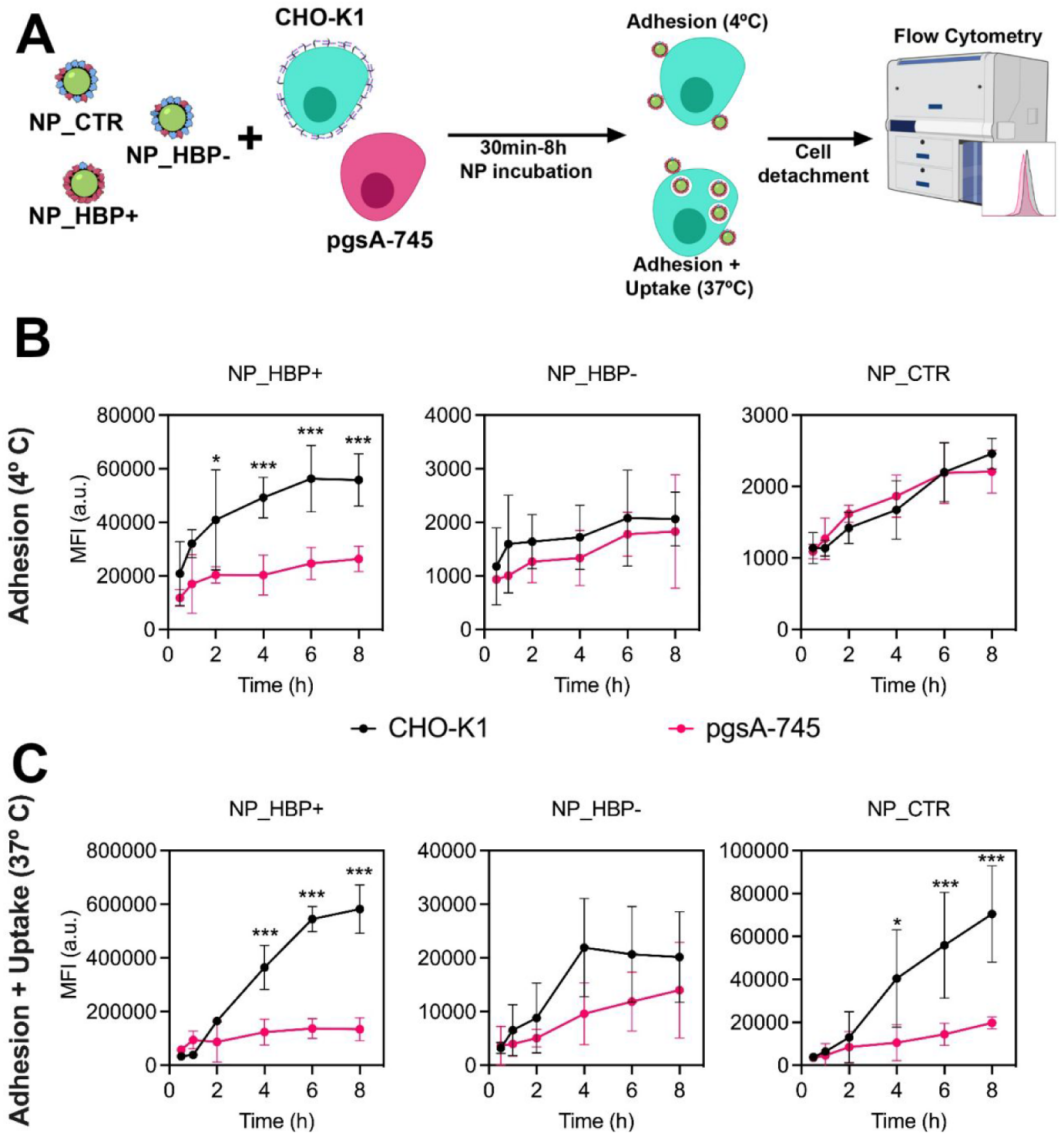
Kinetics of NP surface adhesion and uptake in CHO-K1 and pgsA-745 cells. (A) Schematic overview of the experiments. Cells were exposed to NP_HBP+, NP_HBP– or NP_CTR (50 μg mL^–1^) between 30 min and 8 h at either 4 or 37 °C in serum-free medium, and uptake was quantified by flow cytometry. (B) NP surface adhesion evaluated at 4 °C. (C) NP uptake evaluated at 37 °C. Results represent the mean ± SD of cell fluorescence intensities from three independent replicates. Some illustrations in panel (A) were adapted from the NIAID NIH BioArt collection (bioart.niaid.nih.gov/bioart).^37^.

We first assessed NP–cell interactions at 4 °C (Figure 4B). At this temperature, active endocytosis is inhibited, limiting NP interactions to surface binding.^47^ NP_HBP+ surface adhesion was significantly higher on CHO-K1 cells than on pgsA-745 cells, showing a time-dependent increase that plateaued at approximately 6 h. In contrast, NP_HBP– and NP_CTR exhibited comparable adhesion levels across both cell lines. These results support the notion that NP_HBP+ interacts more strongly with surface GAGs, allowing it to withstand washing steps and accumulate on the CHO-K1 cell surface. As an additional observation, NP_HBP+ showed significantly higher adhesion than NP_HBP– and NP_CTR in both cell lines. While this was expected for CHO-K1, the increased adhesion in pgsA-745 cells may reflect NP_HBP+ binding to residual GAGs, as this mutant retains 3–10% of the total GAGs found in CHO-K1.^42^ Moreover, it may also reflect NP_HBP+ interactions with membrane receptors and/or with sialic acid moieties on glycoproteins, both of which are more accessible in the absence of the GAG barrier.

As a next step, we evaluated NP uptake at 37 °C (Figure 4C). We first note that NP–cell association was markedly higher at 37 °C than at 4 °C, confirming the involvement of energy-dependent endocytosis. For NP_HBP+ and NP_CTR, the uptake difference between CHO-K1 and pgsA-745 increased steadily over time (Figure 4C). We then selected a 4-h time point for further comparisons of uptake among the different NPs. In CHO-K1 cells (Figure 5A), NP_HBP+ exhibited a substantial 10-fold increase in uptake compared with NP_CTR and a 23-fold increase compared with NP_HBP–. A similar pattern was observed in pgsA-745 cells (Figure 5B). The enhanced uptake of NP_HBP+ compared with the other NPs in both cell lines can be attributed to its stronger adhesion to the cell surface, as noted above. To validate the uptake trends observed at 37 °C, we performed laser scanning confocal microscopy. Overall, the confocal results were consistent with the flow cytometry data (Figure 5C). In CHO-K1 cells, NP_HBP+ exhibited stronger intracellular fluorescence than NP_HBP– and NP_CTR, with similar patterns observed in pgsA-745 cells.

**Figure 5:**
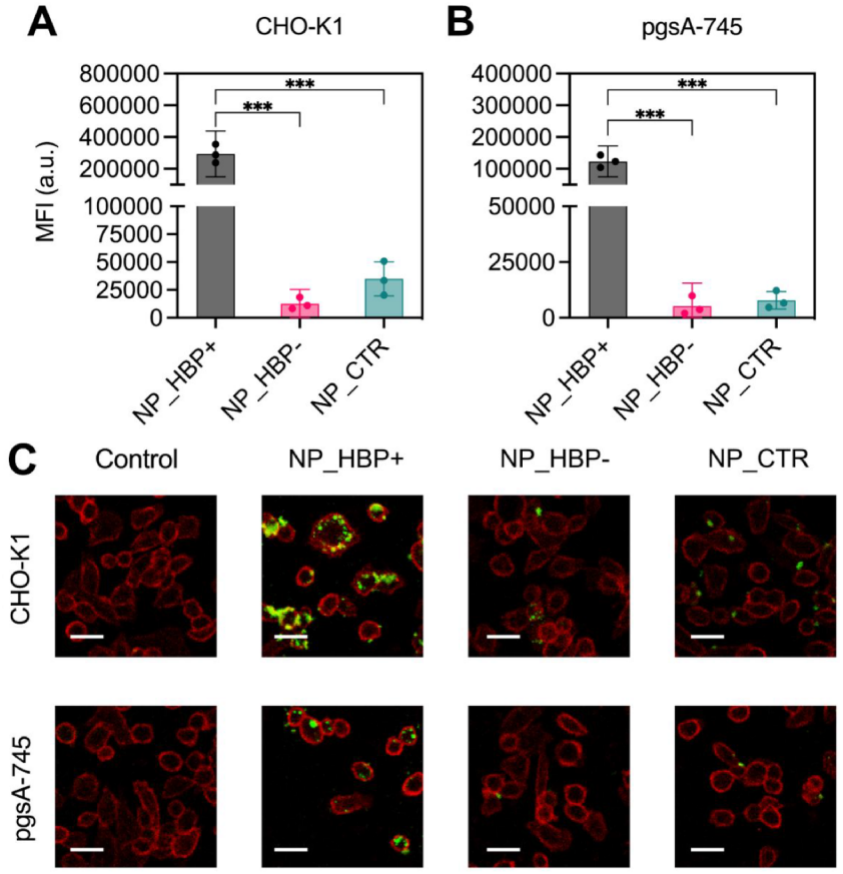
NP uptake in (A) CHO-K1 and (B) pgsA-745 cells. Cells were exposed to NP_HBP+, NP_HBP– or NP_CTR (50 μg mL^– 1^, 4 h, 37 °C, serum-free medium), and uptake was quantified by flow cytometry. Bars represent mean ± SD of cell fluorescence intensities from three independent measurements. (C) Confocal microscopy images of CHO-K1 and pgsA-745 cells following exposure to NPs (50 μg mL^– 1^, 4 h, 37 °C, serum-free medium). After washing, cells were labeled with Alexa Fluor 647-wheat germ agglutinin (WGA) to visualize the plasma membrane. NPs appear in green, while WGA-labeled membranes appear in red. Scale bar, 20 μm.

To further investigate NP uptake, we examined the endocytic mechanisms underlying internalization in CHO-K1 cells. NP_HBP+ utilized both clathrin-mediated endocytosis (CME) and CLIC/GEEC pathways, NP_HBP– relied predominantly on CME, and NP_CTR engaged CME, CLIC/GEEC, macropynocytosis, and lipid raft/cholesterol-dependent pathways (Suppl. Figures S2 and S3).

In summary, NP_HBP+ showed stronger cell-surface adhesion and higher intracellular uptake than NP_HBP– and NP_CTR in both wild-type and mutant cell lines. In the following, we discuss limitations of using the pgsA-745 mutant to understand glycocalyx-mediated NP uptake. We then present additional experiments demonstrating that the strong uptake of NP_HBP+ in CHO-K1 cells is driven by corona–glycocalyx interactions, with consistent trends observed across other corona-coated NPs and cell systems.

### Impact of Corona–Glycocalyx Interactions on NP Uptake

We next focused on the specific role of corona–glycocalyx interactions in mediating NP uptake. A schematic representation of the experimental approach is summarized in Figure 6A. We began by directly comparing NP uptake between CHO-K1 and pgsA-745 cells (Figure 6B). For both NP_HBP+ and NP_CTR, uptake in pgsA-745 cells decreased by ∼ 3-fold, whereas NP_HBP– was not affected. To complement these studies, CHO-K1 cells were enzymatically treated with human heparitinase II (HSase) to remove HS, chondroitinase AC to remove CS, or a combination of both enzymes to shed both HS and CS components. This strategy mitigates potential biases introduced by differences in the endocytic phenotypes of wild-type and mutant cell lines. As shown in Figure 6B, dual enzymatic treatment reduced the cell uptake of both NP_HBP+ and NP_CTR, whereas NP_HBP– uptake was only slightly reduced.

**Figure 6:**
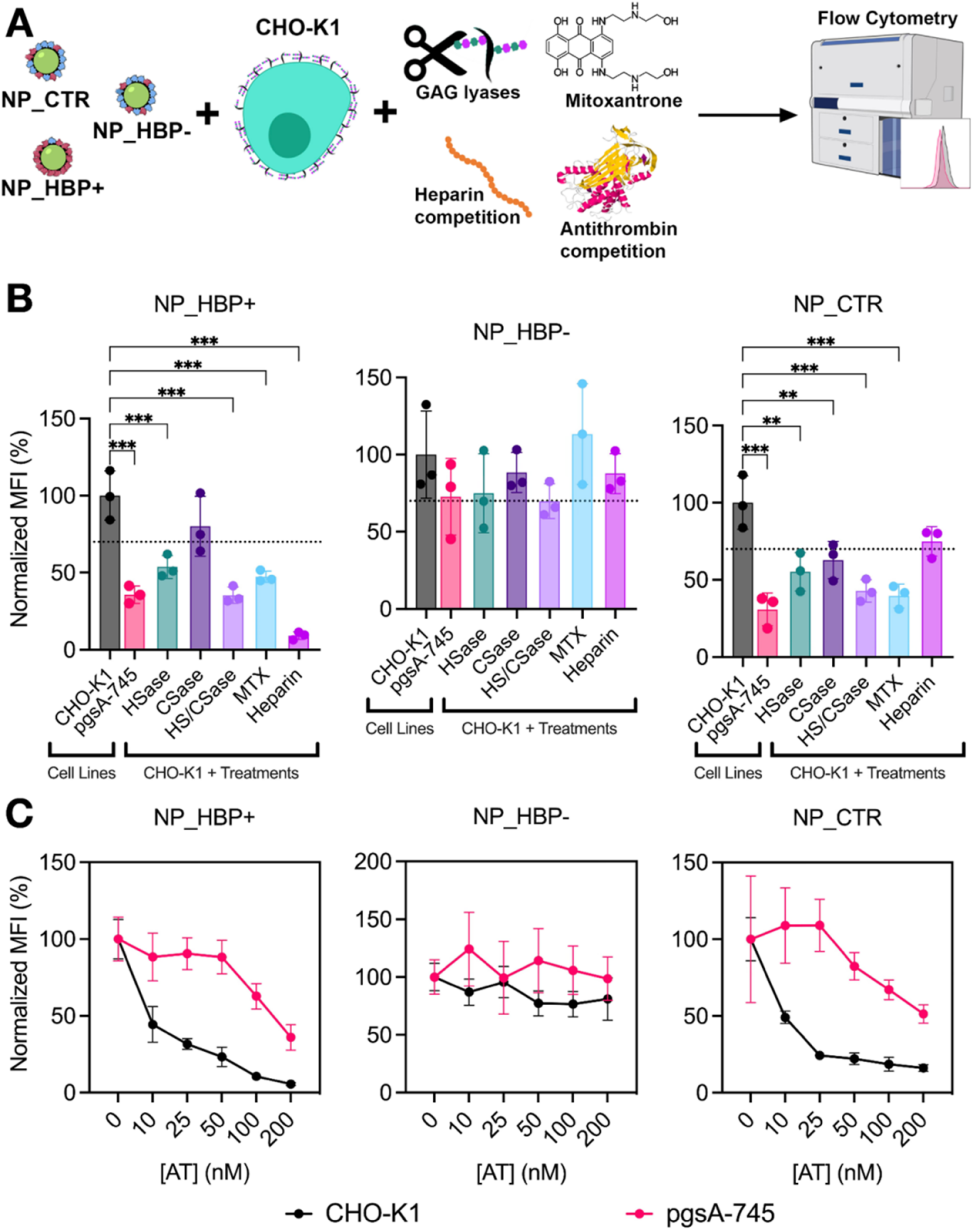
Influence of the cell glycocalyx on the uptake of NP_HBP+, NP_HBP–, and NP_CTR by CHO-K1 cells. (A) Schematic of the experimental design. (B) NP uptake in untreated CHO-K1 cells, pgsA-745 cells, and CHO-K1 cells subjected to enzymatic GAG shedding (HSase and CSase, alone or combined), MTX treatment (50 μM), or heparin competition (100 μg mL^–1^) prior to NP exposure. The dashed line marks the 70% inhibition threshold, beyond which NP uptake is considered reduced. (C) NP uptake in CHO-K1 and pgsA-745 cells pretreated with antithrombin (AT) at the indicated concentrations. In both (B) and (C), cells were exposed to NPs (50 μg mL^–1^, 2 or 4 h, 37 °C, serum-free medium), and uptake was quantified by flow cytometry. Data represent normalized mean ± SD of cell fluorescence intensities from three independent experiments. Some illustrations in panel (A) were adapted from the NIAID NIH BioArt collection (bioart.niaid.nih.gov/bioart).^37^

To further validate these findings, we conducted uptake studies in CHO-K1 cells treated with mitoxantrone (MTX), a compound recently shown to inhibit HSPG-dependent internalization of various cargoes, including cationic GFP, NPs, and SARS-CoV-2.^20,21,48,49^ As depicted in Figure 6B, MTX treatment similarly reduced the uptake of NP_HBP+ and NP_CTR, while NP_HBP– uptake remained unaffected. We also conducted uptake studies in the presence of excess heparin. This strategy aimed to disrupt corona–glycocalyx interactions through heparin binding to the protein corona. The results showed complete inhibition of NP_HBP+ uptake, while NP_HBP– and NP_CTR uptake remained mostly unaffected (Figure 6B). However, it is important to note that heparin binding to the protein corona alters NP surface properties, potentially interfering not only with corona–glycocalyx interactions but also with corona–receptor interactions.

Finally, we implemented an alternative approach to selectively disrupt corona–glycocalyx interactions in CHO-K1 cells without modifying NP properties (as occurs with heparin treatment) or glycocalyx architecture (as occurs in pgsA-745 cells or with enzymatic shedding). This strategy involved performing NP uptake studies in the presence of added AT. We recall that AT binds with high affinity to a small subpopulation of HS containing a specific pentasaccharide motif.^50^ However, beyond this well-characterized interaction, studies have also suggested that AT may associate with other GAG domains and possibly additional regions on the cell surface.^50–200^ Thus, AT was used to block potential glycocalyx binding sites and thereby prevent direct corona–glycocalyx interactions. In CHO-K1 cells, the uptake of NP_HBP+ and NP_CTR decreased in a dose-dependent manner with AT, whereas the uptake of NP_HBP– remained unchanged (Figure 6C). At the highest AT of 200 nM AT, NP_CTR uptake was reduced by 6.7-fold, whereas NP_HBP+ uptake dropped markedly by 20-fold. In pgsA-745 cells, as expected, AT had a much smaller effect on the uptake of NP_HBP+ and NP_CTR (Figure 6C). The fact that AT still inhibited uptake to some extent is consistent with NPs interacting with remaining GAGs and possibly other nonspecific binding sites. To exclude the possibility that the reduced uptake of NP_HBP+ and NP_CTR in CHO-K1 cells was caused by AT interference with endocytic processes, we examined whether AT affected the internalization of endocytic markers. Specifically, we assessed fluorescently labeled transferrin, 70 kDa dextran, and anti-CD44 to evaluate CME, macropinocytosis, and CLIC/GEEC-dependent endocytosis, respectively—the main pathways involved in the internalization of our NPs (Suppl. Figure S2). The results showed that AT had only a minor effect on CME, while it did not affect macropinocytosis or the CLIC/GEEC pathway (Suppl. Figure S4). Thus, the marked reduction in NP uptake in CHO-K1 cells cannot be attributed to AT interference with endocytic pathways. Next, to provide direct evidence that AT binding to surface GAGs inhibits NP_HBP+ interactions with the glycocalyx, we conducted experiments at 4 °C, a condition that allows NP adhesion to the cell surface but prevents endocytosis. We preincubated CHO-K1 and pgsA-745 cells with AT before NP_HBP+ addition at 4 °C. The results confirmed that AT effectively blocked NP_HBP+ adhesion to CHO-K1 cells, reducing it to the levels observed in pgsA-745 cells (Suppl. Figure S5). To exclude the possibility that AT could be inhibiting NP uptake through nonspecific steric effects, we tested NP uptake in the presence of added human serum albumin (HSA). Overall, HSA had minimal effect on NP_HBP+ and NP_CTR uptake in both CHO-K1 and pgsA-745 cells, even at concentrations up to 50 μM (Suppl. Figure S6).

Taken together, the above results indicate that for both GAG-binding NPs, NP_HBP+ and NP_CTR, interactions with the glycocalyx enhances cellular uptake. In contrast, NP_HBP–, which lacks GAG-binding ability, shows minimal dependence on the glycocalyx for uptake.

In closing this part, we note that the impact of corona–glycocalyx interactions on NP uptake varied depending on the experimental approach. For NP_HBP+ in particular, uptake was only ∼ 3-fold lower in enzyme-treated CHO-K1 and GAG-deficient pgsA-745 cells compared to untreated CHO-K1 cells (Figure 6B), whereas competition with AT in CHO-K1 cells caused a substantial 20-fold reduction in uptake (Figure 6C). This discrepancy can be explained by considering the dual role of the glycocalyx in NP–cell interactions and uptake. Specifically, methods involving glycocalyx depletion or shedding not only eliminate the steric and electrostatic barrier functions of the glycocalyx but also remove GAG binding sites for the protein corona. The net outcome may be either an increase or decrease in NP uptake, depending on which factor predominates. In contrast, AT blocks GAG-binding sites while preserving the structural integrity of the glycocalyx, allowing for an evaluation of how corona–glycocalyx interactions may contribute to NP uptake.

### Glycocalyx-Mediated Uptake of NPs Coated with Individual HBPs and Non-HBPs

We next applied an alternative approach to further validate the role of glycocalyx interactions in NP uptake, using simplified coronas composed of HSA supplemented with individual HBPs or non-HBPs. The three most abundant proteins from the NP_HBP– corona (HSA, APOA1, α1AT) were chosen as non-HBPs, while those from the NP_HBP+ corona (APOE, AT, PF4) were selected as HBPs (Figure 7A). For convenience, HDL and VLDL lipoproteins were used to generate coronas enriched in APOA1 and APOE, respectively. DLS analysis confirmed that all HBP-containing NPs interacted with heparin, as evidenced by heparin-mediated NP aggregation, whereas NPs coated with non-HBPs exhibited minimal heparin interactions (Suppl. Figure S7). We note that α1AT actually contains a heparin-binding site;^54^ nevertheless, it behaves as a non-HBP, likely due to its orientation within the protein corona.

**Figure 7:**
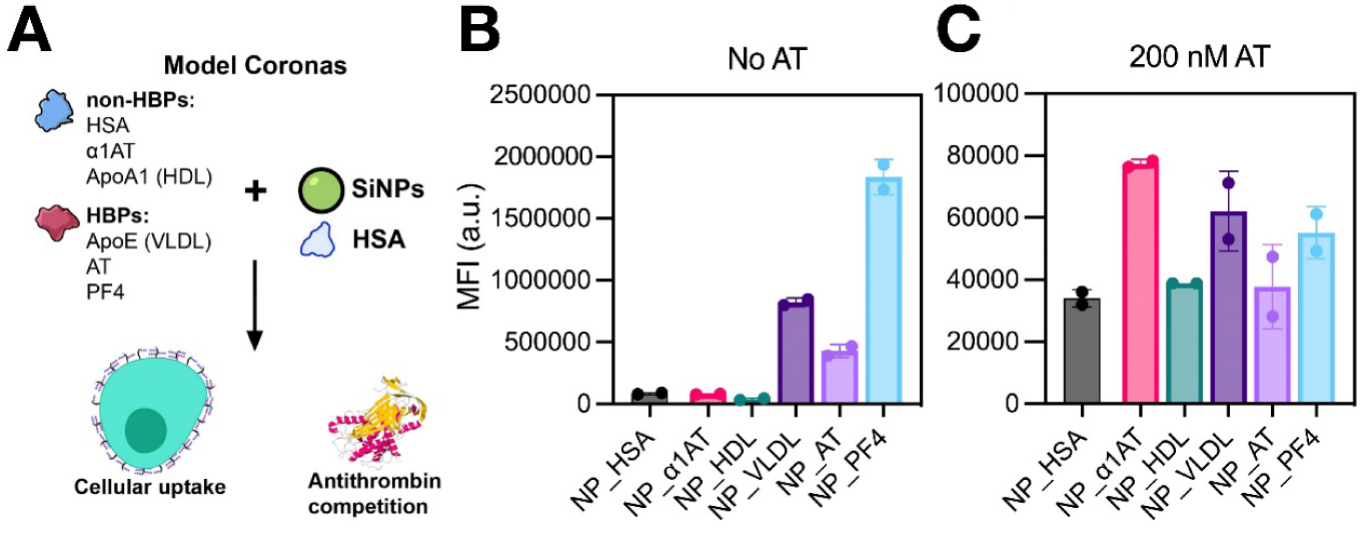
Uptake of model NPs with coronas containing HBPs or non-HBPs by CHO-K1 cells. (A) Schematic of the experimental design. (B) NP uptake in CHO-K1 cells. (C) NP uptake in CHO-K1 cells pretreated with 200 nM AT. Cells were exposed to NPs (50 μg mL^–1^, 4 h, 37 °C, serum-free medium), and uptake was quantified by flow cytometry. Data represent the mean ± SD of cell fluorescence intensities from two independent experiments. Some illustrations in panel (A) were adapted from the NIAID NIH BioArt collection (bioart.niaid.nih.gov/bioart).^37^.

Figure 7B compares uptake levels of the six model NPs in CHO-K1 cells. NPs containing HBPs showed markedly higher uptake than those coated with non-HBPs. NP_AT, NP_VLDL, and NP_PF4 exhibited 5-, 10-, and 22-fold higher uptake, respectively, compared to NP_HSA. These enhancements matched or even surpassed the 10-fold increase previously observed with a fully cationized albumin corona.^20^ To confirm that this enhanced uptake was facilitated by corona–glycocalyx interactions, we blocked GAG-binding sites with AT. Treatment with 200 nM AT reduced uptake of all HBP-containing NPs to levels comparable to non-HBP controls (Figure 7C; full titration curves in Suppl. Figure S8). These findings confirm that coronal HBPs substantially enhance NP uptake through glycocalyx interactions.

Here, the use of simplified coronas enabled additional insights by minimizing confounding effects associated with complex coronas. First, NP_AT and NP_PF4 achieved high uptake despite AT and PF4 lacking identified membrane receptors for their own internalization. This highlights that glycocalyx-mediated NP surface retention alone can drive efficient cellular uptake, independent of specific corona–membrane receptor interactions. Second, NP_VLDL uptake decreased from 10- to only 1.7-fold higher than NP_HSA when glycocalyx interactions were disrupted with AT, suggesting that even strong corona–receptor interactions (APOE–LDLR) contribute little to uptake when the glycocalyx primarily acts as a barrier. This premise, however, requires further examination, as APOE–LDLR interactions were not directly assessed in the present study.

### Influence of Excess Free Proteins on NP Uptake

The uptake experiments described thus far were performed in serum-free medium. To evaluate the impact of excess free protein, we incubated CHO-K1 cells with NP_HBP+, NP_HBP–, and NP_CTR in the presence of 1%, 10%, and 50% (v/v) normal human serum (HS), as well as 10% (v/v) fetal bovine serum (FBS). The 50% HS condition mimicked physiological serum levels, whereas 10% FBS reflected standard cell culture conditions. We found that uptake of NP_HBP– and NP_CTR was substantially reduced under all conditions except 1% HS (Figure 8A). By contrast, a reduction in NP_HBP+ uptake was observed only at the highest HS concentration (50%). Even then, uptake remained significantly higher (by a factor of 30-fold) compared to NP_HBP– and NP_CTR. To gain further insight, we repeated the uptake experiments in pgsA-745 cells. In this GAG-deficient model, the presence of excess protein markedly reduced the uptake of all three NPs, including NP_HBP+ (Figure 8B). These findings highlight the importance of corona–glycocalyx interactions in sustaining efficient NP uptake within complex biofluids.

**Figure 8:**
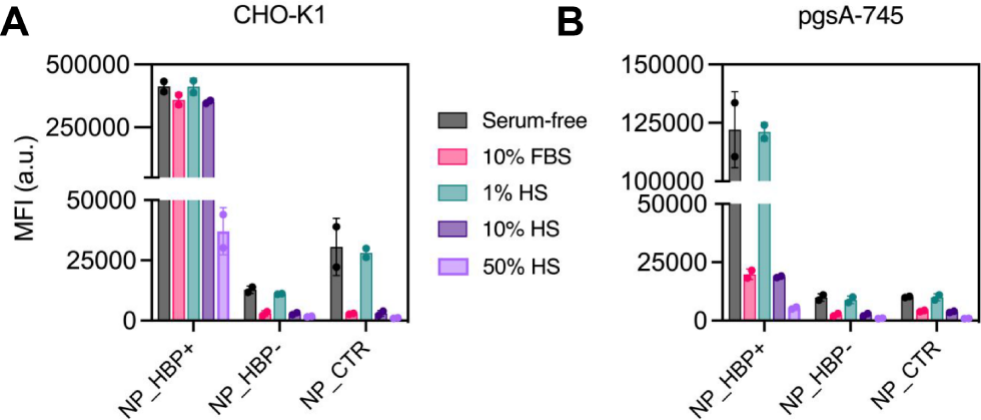
Influence of excess free protein on NP uptake by (A) CHO-K1 and (B) pgsA-745 cells. NP_HBP+, NP_HBP–, or NP_CTR (50 μg mL^–1^) were incubated with the indicated biofluids (see legend) and applied to cells for 4 h at 37 °C. NP uptake was quantified by flow cytometry. Data represent the mean ± SD of cell fluorescence intensities from two independent experiments.

### Glycocalyx-Mediated Uptake of NPs with Physiologically Derived Coronas

We next examined whether the trends observed above extend to more complex and physiologically relevant systems. Specifically, we hypothesized that sera from dyslipidemic individuals, defined here by elevated total cholesterol and triglyceride levels, would yield coronas enriched in apolipoprotein B-100 (APOB) and APOE, which are key apolipoprotein components of low-density lipoprotein (LDL) and very-low-density lipoprotein (VLDL). As both APOB and APOE are well-known HBPs, their enrichment in protein coronas would therefore be expected to enhance uptake via corona–glycocalyx interactions. To test this hypothesis, we collected and pooled serum samples from control and dyslipidemic individuals, further subdividing the dyslipidemic group into two pools based on their total cholesterol and triglyceride levels (Figure 9A). The control pool and dyslipidemic pools 1 and 2 had cholesterol levels of 169, 231, and 258 mg dL^–1^, and triglyceride levels of 107, 116, and 159 mg dL^–1^, respectively. We then coated silica NPs with control and dyslipidemic sera, yielding NP_CT, NP_DL1, and NP_DL2. The resulting corona compositions were characterized by mass spectrometry, with the most abundant proteins listed in Suppl. Table S4. Figure 9B ranks the top 20 proteins, accounting for approximately 80% of the total RPAs, with HBPs highlighted in red. As hypothesized, both NP_DL1 and NP_DL2 coronas (particularly NP_DL2) exhibited higher RPAs for both APOB and APOE compared with NP_CT. NP_DL1 and NP_DL2 coronas also contained additional HBPs, including APO­(a) and PF4. Overall, HBPs within the top 20 proteins accounted for cumulative RPAs of 13.5% and 15.3% in NP_DL1 and NP_DL2, respectively, versus only 6.4% in NP_CT. Consistent with their greater HBP content, NP_DL1 and NP_DL2 interacted with heparin, as measured by DLS and SPR, whereas NP_CT exhibited no significant interactions (Suppl. Figure S9).

**Figure 9:**
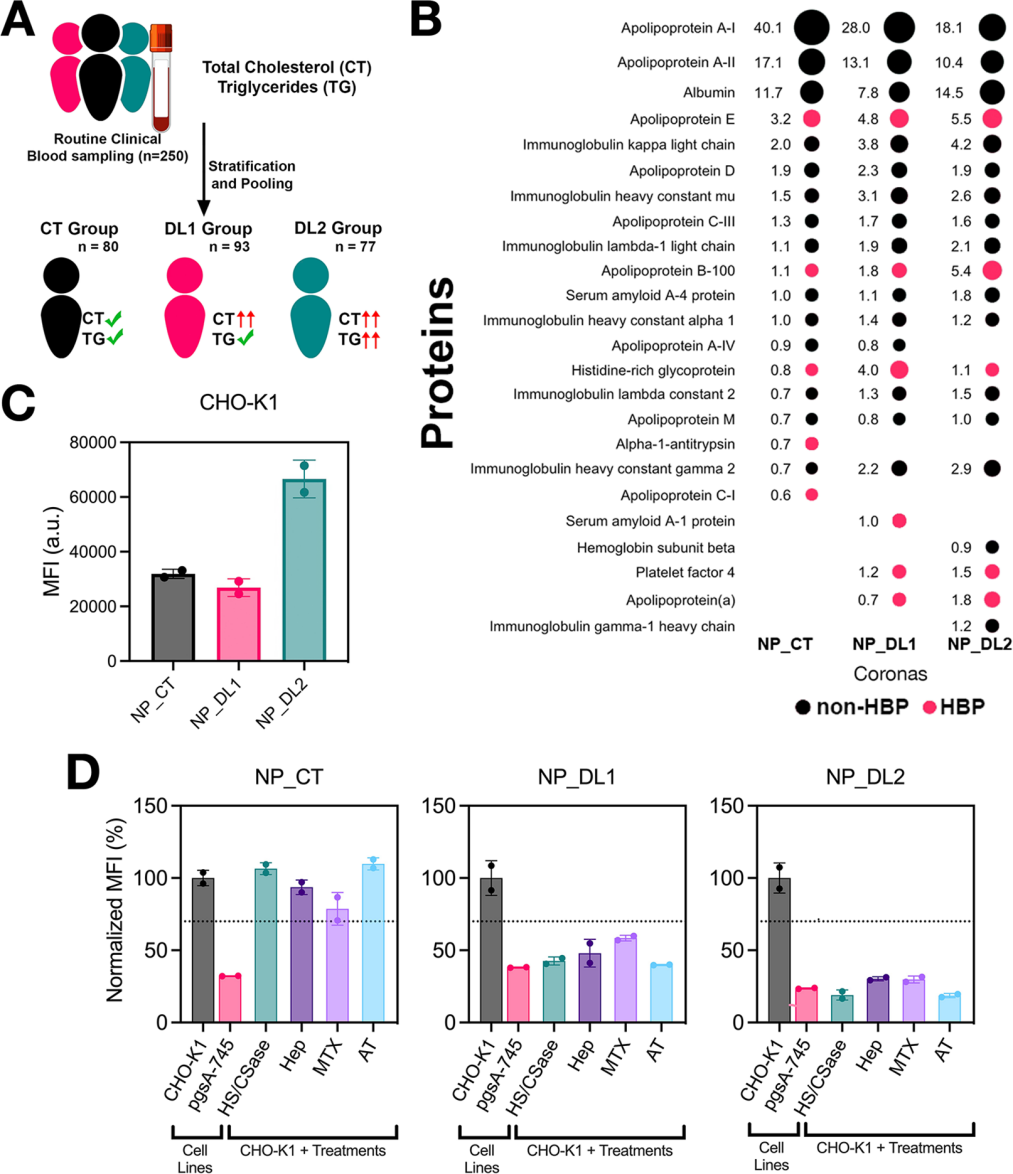
Assessment of glycocalyx-mediated uptake of NPs coated with physiologically derived protein coronas. (A) Schematic overview of the CT, DL1, and DL2 biofluids used for the preparation of corona-coated NPs. (B) Top 20 corona proteins identified by mass spectrometry for each NP type, with RPA values indicated. Proteins reported in the literature as HBPs are highlighted in red. (C) NP uptake in CHO-K1 cells. (D) NP uptake in CHO-K1 cells, pgsA-745 cells, or CHO-K1 cells treated with HSase plus CSase, MTX (50 μM), heparin (100 μg mL^–1^), or AT (400 nM) prior to NP exposure. In (C) and (D), cells were exposed to NPs (50 μg mL^–1^, 2 or 4 h, 37 °C, serum-free medium), and uptake was quantified by flow cytometry. Data represent mean (or normalized mean) ± SD of cell fluorescence intensities from two independent experiments. The dashed line marks the 70% uptake threshold, beyond which NP uptake is considered reduced. Some illustrations in panel (A) were adapted from the NIAID NIH BioArt collection (bioart.niaid.nih.gov/bioart).

Having characterized the corona compositions and NP interactions with GAGs, we next evaluated NP uptake in CHO-K1 cells. NP_DL2 showed approximately 2-fold higher uptake than NP_CT, whereas NP_DL1 uptake was similar to NP_CT (Figure 9C). To probe the contribution of corona–glycocalyx interactions, we compared uptake in pgsA-745 versus CHO-K1 cells. All NPs showed reduced uptake in pgsA-745, with the strongest decrease observed for NP_DL2 (Figure 9D). However, since this mutant line may display altered endocytosis unrelated to GAGs, we repeated the experiments in CHO-K1 cells treated with glycosidic enzymes, heparin, MTX, and AT. In all cases, NP_CT uptake was unaffected, whereas NP_DL1 and NP_DL2 uptake were significantly reduced, particularly NP_DL2 (Figure 9D).

Collectively, these results confirm the critical role of corona–glycocalyx interactions in promoting NP uptake, even for complex, physiologically relevant coronas. Interestingly, these findings also suggest that glycocalyx interactions could be leveraged to modulate NP uptake for therapeutic purposes, taking advantage of altered coronas arising from specific physiological states.

### Glycocalyx-Mediated NP Uptake in Human Cell Lines

Having used CHO cells as a well-established GAG-relevant model, we next asked whether glycocalyx-mediated NP uptake extends to human cell lines. Given the relevance of NPs in cancer therapy, we initially focused on MDA-MB-231, a triple-negative breast cancer model. Experiments at 4 °C revealed significantly greater cell-surface adhesion for NP_HBP+ compared with NP_HBP– and NP_CTR (Suppl. Figure S10A). At 37 °C, NP_HBP+ also displayed markedly higher uptake (Suppl. Figure S10B). To probe the role of the glycocalyx, we compared uptake in control cells versus cells pretreated with AT, finding that pretreatment inhibited NP_HBP+ uptake, whereas NP_HBP– and NP_CTR uptake remained unaffected (Suppl. Figure S10C). We also examined NPs coated with simplified coronas composed of HSA mixed with either HBPs or non-HBPs to minimize confounding effects associated with complex coronas. We found that HBP-containing NPs (NP_AT, NP_VLDL, NP_PF4) exhibited much higher uptake than their non-HBP counterparts, confirming that HBPs within the corona can substantially enhance uptake (Suppl. Figure S10D). As a further test, we assessed NP uptake and the contribution of glycocalyx interactions in human umbilical vein endothelial cells (HUVECs), representing an endothelial model, and in HeLa cells as an additional cancer model. The results again recapitulated the uptake patterns observed in CHO-K1 and MDA-MB-231 (Suppl. Figure S11).

We finally tested whether similar trends would be observed with physiologically derived coronas. MDA-MB-231, HUVEC, and HeLa cells were used as models. NP_DL2 exhibited higher uptake than NP_CT in HUVEC and HeLa, although uptake levels were comparable in MDA-MB-231 cells (Suppl. Figure S12B). Moreover, AT competition strongly reduced NP_DL2 uptake in HUVEC and HeLa, while NP_CT uptake remained unaffected in these cells (Suppl. Figure S12B). Overall, these findings mirror those observed in CHO-K1 cells, providing further evidence that corona–glycocalyx interactions can drive NP uptake even for physiologically relevant coronas.

### Model for Glycocalyx-Mediated NP Uptake

Our proposed model for glycocalyx-mediated NP uptake is summarized in Figure 10, building on both our current findings and previous work. As new studies expand our understanding, the model may be refined. Below, we outline its key features.

**Figure 10:**
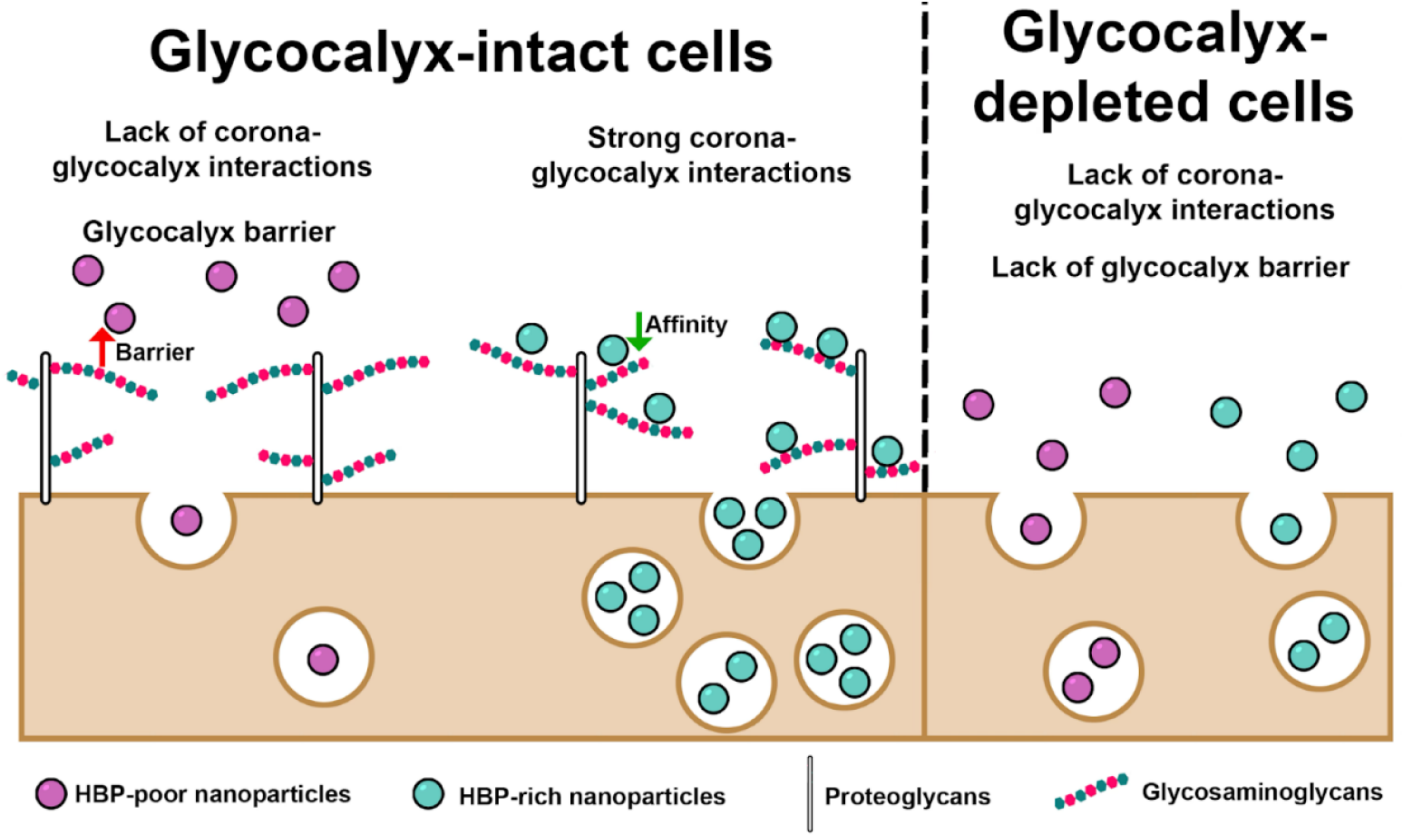
Schematic model illustrating the role of the glycocalyx in regulating NP uptake.

(1) The efficient uptake of corona-coated NPs by cells with a normal glycocalyx depends on two critical conditions: (i) favorable corona–glycocalyx interactions and (ii) activation of productive cell entry pathways. If the first condition is unmet, uptake is drastically reduced, as the glycocalyx fails to retain NPs at the cell surface and instead acts as a steric and/or electrostatic barrier. If the second condition is not fulfilled, NPs may accumulate within the glycocalyx but undergo limited internalization.
(2) Strong corona–glycocalyx interactions can be mediated by classic HBPs within the protein corona (e.g., AT, APOE, PF4). These strong interactions promote more sustained NP accumulation at the cell surface and greater uptake compared to weaker interactions. The magnitude of the uptake enhancement can match, or even exceed, levels observed with cationic or surface-functionalized NPs.^20,21,55–57^

(3) In glycocalyx-depleted cells, NPs reach the cell surface unhindered by the glycocalyx, facilitating interactions between corona proteins and membrane receptors that can activate entry pathways. Indeed, for highly negatively charged or PEGylated NPs that lack GAG-binding properties, glycocalyx depletion has been shown to enhance uptake.^20–22,24,27^ However, for GAG-binding NPs, the benefit of glycocalyx depletion may be offset by the loss of glycocalyx-mediated NP retention at the cell surface, ultimately leading to reduced overall uptake.

Future studies should now aim to expand on the current model by exploring diverse glycocalyx structures, corona compositions, and NP properties, to refine our understanding of the glycocalyx’s complex role in NP uptake.

## Conclusions

As NPs approach the cell surface, they encounter the glycocalyx, a dense, negatively charged meshwork of GAGs and other sugar-rich molecules. The glycocalyx can modulate the uptake of corona-coated NPs through multiple simultaneous mechanisms, including steric hindrance, electrostatic interactions, and binding to corona proteins. Here, we uncovered that corona–glycocalyx interactions play a much greater role in NP uptake than previously recognized.

We prepared NPs coated with plasma-derived coronas that were either enriched (NP_HBP+) or depleted (NP_HBP−) in HBPs. We found that NP_HBP+ exhibited strong interactions with GAGs, resulting in sustained NP accumulation at the cell surface and markedly enhanced uptake. Blocking binding sites within the glycocalyx—while preserving its barrier function—led to a pronounced reduction in NP_HBP+ uptake. In contrast, NP_HBP– did not interact with GAGs, resulting in lower uptake that was also unaffected by the glycocalyx. These findings were reproduced across four different cell models. Consistent trends were also observed with simplified coronas containing selected HBPs or non-HBPs, as well as with physiologically derived coronas from normal or dyslipidemic individuals. Importantly, our results revealed that it is not merely the presence of HBPs in the corona, but rather their ability to interact with GAGs, that determines uptake.

Notably, observations with HBP-rich NPs are reminiscent of the behavior of some endogenous NPs (lipoproteins). Specifically, APOE, which is found in lipoprotein remnants, mediates their binding to HSPGs and facilitates uptake by hepatocytes.^33,34^ Similarly, when associated with oxidized LDL, PF4 promotes binding to HSPGs and enhances uptake by vascular macrophages.^58^

Our results also have implications for the therapeutic use of NPs. *In vivo*, HBP content in protein coronas may vary with physiological state, which could lead to substantial differences in cell-surface adhesion, uptake, and ultimately biodistribution and clearance across individuals. In terms of NP design, engineering NP surfaces to engage GAGs in target tissues could provide a more effective way to boost uptake and therapeutic efficacy than relying solely on NP–membrane receptor interactions. Indeed, receptor-targeting strategies alone may underperform if the glycocalyx acts primarily as a barrier rather than a facilitator of NP internalization.

In summary, our findings identify the glycocalyx as a recognition platform for coronal HBPs that can actively drive NP uptake, independent of specific corona–membrane receptor interactions or NP charge. This suggests that the prevailing paradigm in nanomedicine—centered on engineering the biomolecular corona to optimize interactions with membrane receptors—should evolve to encompass the strategic optimization of glycocalyx interactions. This expanded approach could enable the fine-tuning of cellular uptake and promote more efficient and selective drug delivery to both normal and disease-altered glycocalyx environments, such as in cancer or atherosclerosis.

## Materials and Methods

### Chemicals

Fluorescent silica NPs (Ex/Em = 494/521 nm) were from Kisker Biotech (Steinfurt, Germany). Human plasma, anticoagulated with sodium citrate, was obtained from the Charitable Association of Blood Collection (COLSAN, São Paulo, Brazil). AT and PF4 were from Enzyme Research Laboratories (South Bend, USA). HDL and VLDL were from Innovative Research (Novi, USA). HSA, α1AT, chlorpromazine, 5-(N-ethyl-*N*-isopropyl)­amiloride (EIPA), nystatin, dynasore, 7-ketocholesterol, heparin, MTX, FITC-labeled anti-CD44, F-12, DMEM and RPMI culture media were from Sigma-Aldrich (São Paulo, Brazil). Pierce Quantitative Colorimetric Peptide Assay, Micro-BCA Protein Assay Kit, Alexa Fluor 488-labeled transferrin, FITC-labeled 70 kDa Dextran, and Alexa Fluor 746-wheat germ agglutinin (WGA) were from Thermo Fisher Scientific (São Paulo, Brazil). RapiGest SF Surfactant was from Waters (São Paulo, Brazil). HSase was from R&D Systems (USA), and CSase was prepared from *Flavobacterium heparinum* as reported elsewhere.^59^

### Human Plasma Fractionation

Approximately 200 mL of pooled human plasma were initially dialyzed using 3 kDa MWCO Amicon tubes (4000g, 30 min) against buffer A (10 mM phosphate buffer, pH 7.4, 0.25 M NaCl). For heparin-affinity chromatography, a 52 mL Heparin Sepharose Fast Flow column (Cytiva, Marlborough, USA) was connected to an AKTApure (Cytiva) protein purification system. Following equilibration with buffer A, the plasma was circulated through the column at 2.5 mL min^–1^ for five complete cycles. The column was washed with buffer A (5 mL min^–1^, 5 column volumes), and the flow-through was combined with the unbound plasma to generate the HBP– biofluid. Bound HBPs were then eluted with buffer B (10 mM phosphate buffer, pH 7.4, 2 M NaCl) at 2 mL min^–1^ (5 column volumes). This HBP+ biofluid was desalted into 150 mM NaCl using a Sephadex G-25 column (Cytiva), while the HBP– and CTR biofluids were dialyzed against 150 mM NaCl. The resulting biofluids were adjusted to a uniform protein concentration prior to use.

### Serum Collection

Blood sampling was approved by the research ethics committees of the Universidade Federal de São Paulo (UNIFESP) and the Centro Universitário Faculdade de Medicina do ABC (FMABC) (approval # 48006721.2.0000.5505). Blood samples were collected from individuals undergoing routine clinical testing at FMABC. For each donor, total cholesterol and triglyceride levels were measured using automated clinical enzymatic colorimetric assays (Cobas 8000 analyzer, Roche). Serum samples were then pooled according to the criteria described in the text, yielding pools from 80 control, 93 dyslipidemic group 1, and 77 dyslipidemic group 2 sera. After pooling, average total cholesterol and triglyceride levels were determined in accordance with good clinical laboratory practice. The large pool size minimized interindividual variability, allowing us to focus specifically on differences in lipid content.

### Protein Corona Formation and Characterization

Bare silica NPs (250 μg mL^–1^) were incubated with HBP+, HBP– or CTR biofluids (1 mg mL^–1^ protein) for 24 h at 4 °C under mild agitation. Unbound proteins were removed by three consecutive cycles of centrifugation (15000g, 20 min, 15 °C) and wash in PBS (10 mM phosphate buffer, pH 7.4, 150 mM NaCl). After the last cycle, the pellet was resuspended in the appropriate medium for subsequent experiments. To quantify the adsorbed protein mass, the pellet from the last centrifugation step was resuspended in 100 μL of 50 mM NH_4_HCO_3_ buffer containing 25 μL of 0.1% RapiGest and incubated at 80 °C for 15 min to detach proteins from the NPs. A final centrifugation was performed to remove the NPs, and the supernatant was collected for analysis. Protein concentration was then determined using the Micro-BCA assay. NP size and surface charge were determined by DLS and ZP measurements using a Zetasizer Nano ZS (Malvern Instruments, UK). Measurements were performed at 25 °C with NPs (250 μg mL^–1^) dispersed in ultrapure water. NP protein coronas were also prepared from control and dyslipidemic sera using the same procedure as above, except that NPs were incubated at a protein concentration of 10 mg mL^–1^. Protein coronas were finally prepared using individual HBPs or non-HBPs. To better mimic physiological conditions in which HSA is the predominant plasma protein, the model coronas were prepared with a 10-fold excess of HSA. Bare silica NPs (250 μg mL^–1^) were incubated for 24 h at 4 °C with HSA (1 mg mL^–1^) or with HSA mixed with AT, PF4 or α1AT at a 10:1 mass ratio. Additional NPs were prepared using HSA mixed with VLDL or HDL (10:1) to generate surfaces enriched in APOE or APOA1, respectively. After incubation, unbound proteins were removed, and NPs were recovered as described above. In all cases, fluorescence measurements confirmed that NP fluorescence remained unchanged after corona formation.

### Mass Spectrometry Analysis

Adsorbed proteins were stripped from the NP surface as described above. For protein digestion, samples were first reduced with 5 mM DTT at 60 °C for 30 min, then alkylated with 15 mM IAA at 25 °C for 30 min in the dark. Trypsin was added at a 1:100 enzyme-to-protein ratio, and digestion was carried out overnight. The reaction was quenched with 5% TFA, and samples were subsequently dried. Peptides were fractionated using StageTip C18 columns to remove excess salts and then quantified using the Pierce Quantitative Colorimetric Peptide Assay. Data acquisition was performed as detailed in the Supporting Information. For data analysis, the .raw files were processed and quantified in Progenesis QI for proteomics (PQIP, Nonlinear Dynamics) with automatic alignment and peak picking. The mgf file was imported in Peaks Studio X+ software (BSI, Bioinformatics Solutions Inc.) for de novo and database–based analysis. Database search was performed using the *Homo sapiens* database (20,641 sequences, downloaded from UniProt on April 22nd, 2025), with a maximum false discovery rate of 1%. Relative quantification was performed using the average signal response of the three most intense tryptic peptides of each protein.^60^

### NP–Heparin Interactions

NP–heparin interactions were evaluated using DLS. NPs (250 μg mL^–1^) were incubated with increasing concentrations of heparin in PBS, and heparin-induced NP aggregation was monitored at 25 °C. The interactions were further assessed using SPR on a Biacore T-200 system (Cytiva). Biotinylated heparin was immobilized on a streptavidin-coated sensor chip at a density of 600 response units (RU). NPs, prepared in PBS at concentrations ranging from 0.15 nM to 10 nM, were flowed over the sensor surface at 60 μL min^–1^. Association and dissociation phases were monitored for 200 s and 900 s, respectively. After each run, the surface was regenerated using 0.01% sodium dodecyl sulfate in water, followed by 2 M NaCl in water (60 s injection at 30 μL min^–1^). Bulk refractive index variations were corrected by subtracting the reference channel response. For NP_HBP+ and NP_CTR, the resulting data were analyzed using EVILFIT software, applying a model based on continuous distributions of equilibrium and kinetic rate constants.^61^

### Cells

CHO-K1 and pgsA-745 cells (kindly provided by Dr. Esko, University of California, San Diego, USA) were cultured in Ham’s F-12 Nutrient Mix supplemented with 10% (v/v) FBS and 1% (v/v) penicillin/streptomycin, and maintained in a humidified atmosphere at 37 °C with 5% CO_2_. MDA-MB-231 and HeLa cells were cultured in Dulbecco’s Modified Eagle Medium (DMEM), and HUVEC cells in RPMI 1640, both supplemented as above. All experiments were performed using low-passage cells (passages 1–10 for CHO and HUVEC; 1–20 for HeLa; 1–8 for MDA-MB-231).

### Cellular Uptake

NP surface adhesion and internalization were first investigated in CHO-K1 and pgsA-745 cells. Cells were plated at a density of 10^5^ cells per well in a 24-well plate and maintained in complete medium for 16 h. Following this period, cells were washed and incubated with fresh serum-free medium containing NP_HBP+, NP_HBP–, or NP_CTR (50 μg mL^–1^) at both 4 and 37 °C for time points ranging from 30 min to 8 h. After incubation, cells were washed with ice-cold PBS and analyzed on a BD Accuri C6 flow cytometer (BD Biosciences, USA), with gating to exclude debris and doublets, and a minimum of 10,000 events recorded per sample. For confocal microscopy, cells were incubated with NPs (50 μg mL^–1^) in serum-free medium for 4 h at 37 °C, extensively washed with ice-cold PBS, then stained with WGA-A647 (10 μg mL^–1^) in phenol red-free medium for 10 min at 4 °C. Imaging was performed using a Leica SP2 confocal microscope (Leica Microsystems, Germany).

To determine the mechanisms of cellular uptake, CHO-K1 cells were pretreated with chlorpromazine (40 μM), EIPA (300 μM), nystatin (25 μM), dynasore (7.5 μM), or 7-ketocholesterol (50 μM) in serum-free medium for 30 min at 37 °C prior to NP administration. Afterward, NPs (50 μg mL^–1^) were added to the wells and incubated for an additional 4 h. Cells were then washed as described above and analyzed by flow cytometry.

To investigate the role of the glycocalyx on NP uptake, CHO-K1 cells were pretreated with HSase (1 μL mL^–1^), CSase (1 μL mL^–1^), or a combination of both enzymes in serum-free medium for 2 h at 37 °C; these incubation conditions were validated in our previous work.^20,21^ Afterward, NPs (50 μg mL^–1^) were added to the wells and incubated for an additional 2 h. In another experiment, cells were preincubated with heparin (100 μg mL^–1^) in serum-free medium for 30 min, followed by NP incubation for an additional 2 h. In a separate experiment, cells were pretreated with MTX (50 μM) in serum-free medium for 30 min prior to NP addition for 2 h. Finally, cells were incubated with AT (1–200 nM) in serum-free medium for 30 min before NP addition for an extra 4 h at 37 °C. After each incubation, cells were washed and analyzed by flow cytometry. To evaluate the effect of excess free protein, NPs were dispersed in 1%, 10%, or 50% HS, as well as 10% FBS, then applied to CHO-K1 and pgsA-745 cells for 4 h at 37 °C. Cells were then washed and analyzed by flow cytometry. Uptake experiments were also performed in CHO-K1 cells using NPs coated with control or dyslipidemic sera, as well as with selected HBPs or non-HBPs, as described in the main text and following similar procedures as above. To expand the analysis beyond CHO-K1 cells, NP uptake experiments were also performed using MDA-MB-231, HeLa and HUVEC cells, as described in the main text and using similar procedures as detailed above.

### Statistics

For mass spectrometry analysis of the protein corona, samples were analyzed in triplicate, and RPA values were reported as the average of the three measurements. For NP characterization by DLS and ZP, results were presented as the mean ± SD from triplicate measurements. For NP–heparin interaction analysis by DLS, representative size distribution plots were shown to facilitate comparison across NP types and treatment conditions. For SPR analysis, three immobilization levels were tested with similar trends, and the condition with the highest response was selected for analysis and presentation. For flow cytometry-based uptake experiments, results were plotted as the mean (or normalized mean) ± SD of median cell fluorescence intensity distributions. Sample flow cytometry histograms are provided in the Supplementary Figures, with those corresponding to Figure 5–9 in the main text compiled in Suppl. Figure S13. Each cell uptake experiment was tested in two or three independent experiments to ensure reproducibility. When three experiments were available, one-way ANOVA followed by Tukey’s or Levenne’s post hoc test (with *p < 0.05, **p < 0.01, ***p < 0.001) was applied after confirming that data met the assumptions of normality and homogeneity of variances. For endocytosis inhibition and glycocalyx cleavage or competition assays, a 70% inhibition threshold was defined, below which NP uptake was considered reduced.^62^ For confocal microscopy, experiments were performed twice to confirm reproducibility and representative images were presented.

## Supplementary Material



## Notes

Supplementary Methods, LC-MS/MS methodology; Figure S1, SPR results and analyses; Figure S2, NP uptake mechanisms in CHO-K1 cells; Figure S3, inhibition of CLIC-GEEC uptake pathway; Figure S4, effect of AT on cell uptake of endocytic markers; Figure S5, NP_HBP+ surface adhesion; Figure S6, NP uptake in the presence of HSA; Figure S7, DLS analysis of NP–heparin interactions; Figure S8, uptake of model NPs in the presence of AT; Figure S9, characterization of NP–heparin interactions by DLS and SPR; Figure S10, NP uptake in MDA-MB-231 cells; Figure S11, NP uptake in HUVEC and HeLa cells; Figure S12, cell uptake of NPs coated with physiologically derived protein coronas; Figure S13, sample flow cytometry histograms (PDF)
