# Supplementary material for: Heparin-Binding Proteins in the Nanoparticle Corona Enhance Cellular Uptake through Glycocalyx Interactions
Source: ACS Nano. 2025 Dec 16;19(51):42964–78. doi: 10.1021/acsnano.5c19714 (PMC12756911; doi:10.1021/acsnano.5c19714)
Supplement: Supplementary file 1 [file nn5c19714_si_001.pdf]

Supplementary Information

Heparin-Binding Proteins in the Nanoparticle Corona Enhance Cellular Uptake Through Glycocalyx Interactions

Paulo H. Olivieri Jr.,[1] Jackelinne Y. Hayashi,[1] Ricardo J.S. Torquato,[1] André F. Lima,[1] Thayza P. Pereira,[1] Ismael F. Lima,[2] Fernando L.A. Fonseca,[3,4] Leo K. Iwai,[2] Helena B. Nader,[1] Alexandre K. Tashima,[1] Giselle Z. Justo,[1,*] Alioscka A. Sousa[1,*]

[1] Department of Biochemistry, Federal University of São Paulo (UNIFESP), São Paulo, SP 04044-020, Brazil

[2] Laboratory of Applied Toxinology, Center of Toxins, Immune-response and Cell Signaling LETA/CeTICS, Butantan Institute, São Paulo, SP 05503-900, Brazil

[3] Pharmaceutical Sciences Department, Federal University of São Paulo (UNIFESP), Diadema, SP 09913-030, Brazil.

[4] Clinical Laboratory Department, Faculty of Medicine of ABC (FMABC), Santo Andre, SP 09060-650, Brazil

*Corresponding authors: giselle.zenker@unifesp.br; alioscka.sousa@unifesp.br

SUPPLEMENTARY METHODS

Liquid chromatography-tandem mass spectrometry (LC-MS/MS)

For NP_HBP+, NP_HBP−, and NP_CTR, LC-MS/MS data were acquired in the DDA mode in a Orbitrap Fusion Lumos mass spectrometer (Thermo Scientific) coupled to an Easy-nLC 1200 UHPLC (Thermo Scientific). Briefly, peptides were loaded to a trap column (Acclaim PepMap 100, C18, 3 μm, 75 μm × 2 cm, nanoViper, Thermo Scientific) with 8 μL of solvent A (0.1% formic acid) at 500 bar, and eluted onto a C18 column (Acclaim PepMap RSLC, C18, 2 μm, 75 μm × 15 cm, nanoViper, Thermo Scientific). Peptides were eluted using a linear gradient of 5−28% solvent B (80% acetonitrile in 0.1% formic acid) for 80 min followed by a linear gradient of 28−40% B for 10 min at 300 nL/min. The nanospray Flex NG ion source was operated in positive ESI mode with capillary temperature at 300 °C and S-Lens RF level at 30%. A full MS scan was followed by data-dependent MS2 scans in a 3 s cycle time. Precursor ions selected for MS2 were excluded for subsequent MS2 scans for 40 s. Precursor ions were fragmented by HCD with a normalized collision energy of 30%. The resolution for the full scan mode was set as 120,000 (at m/z 200) and the automatic gain control (AGC) target at 5×10^5. The m/z range 350−1550 was monitored. Each full scan was followed by a data-dependent MS2 acquisition with a resolution of 30,000 (at m/z 200), maximum fill time of 54 ms, and isolation window of 1.2 m/z. Advanced Peak Determination (APD) and RunStart EASY-ICTM were enabled to improve precursor identification and mass calibration.

For NP_CT, NP_DL1, and NP_DL2, peptide samples were resuspended in 0.1% formic acid and analyzed on an Orbitrap Exploris 480 mass spectrometer (Thermo Fisher Scientific, Bremen, Germany) coupled to a Vanquish Neo nanoLC liquid chromatographer (Thermo Fisher Scientific) equipped with a NanoSpray Easy Flex ion source. Chromatographic separation was achieved on a nanoLC Acclaim PepMap NEO analytical column (150 mm × 75 μm, 2 μm) with an Acclaim PepMap 100 C18 trap column (20 mm × 75 μm, 3 μm), both from Thermo Fisher Scientific. Peptide extracts (0.125 μg) were eluted using a gradient from 5-30% buffer B (90% acetonitrile, 0.1% formic acid) in buffer A (0.1% formic acid in water) over 75 min, 30-40% buffer B for 7 min, and 40-99% buffer B for 8 min at a flow rate of 300 nL/min. The electrospray source operated

at 2.1 kV. The mass spectrometer was equipped with a FAIMS (Field Asymmetric Ion Mobility Spectrometry) interface for gas-phase ion separation and operated in data-independent acquisition (DIA) mode. Full MS scans were acquired at a resolution of 60,000 over an m/z range of 400–900, using 4 m/z isolation windows (total of 125 windows). The Automatic Gain Control (AGC) target was set to 300% for MS1 and 1000% for MS/MS scans, both with 50 ms injection times. Fragmentation was carried out with normalized collision energies of 28% and 32%. MS/MS spectra were acquired with FAIMS compensation voltages of –45 V and –60 V, at a resolution of 30,000 and a maximum injection time of 50 ms.

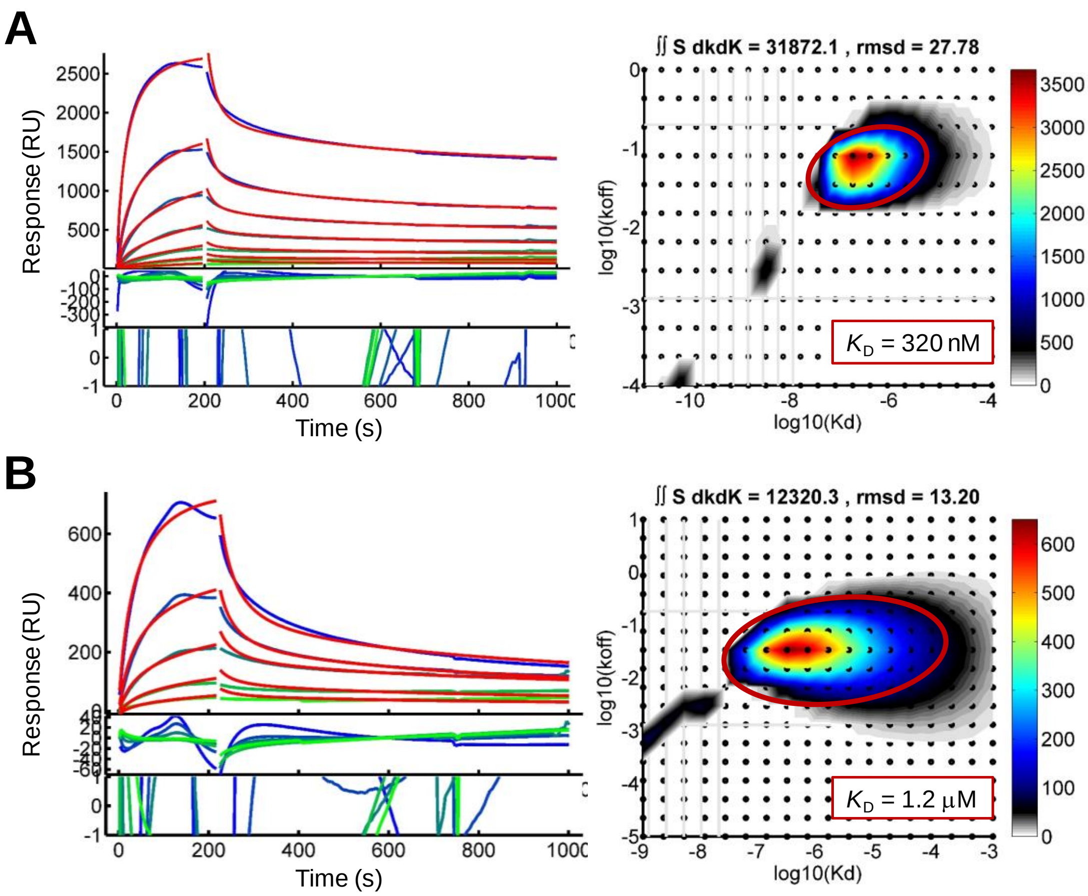

Suppl. Figure S1. Quantitative SPR analysis of (A) NP_HBP+ and (B) NP_CTR binding to heparin using a continuous surface-site distribution model. Left: experimental traces (green and blue lines), fitted curves (red lines), and residuals. Right: corresponding affinity and dissociation rate constant distributions. In each case a major single binding site is identified (red oval), with the extracted K_D value indicated.

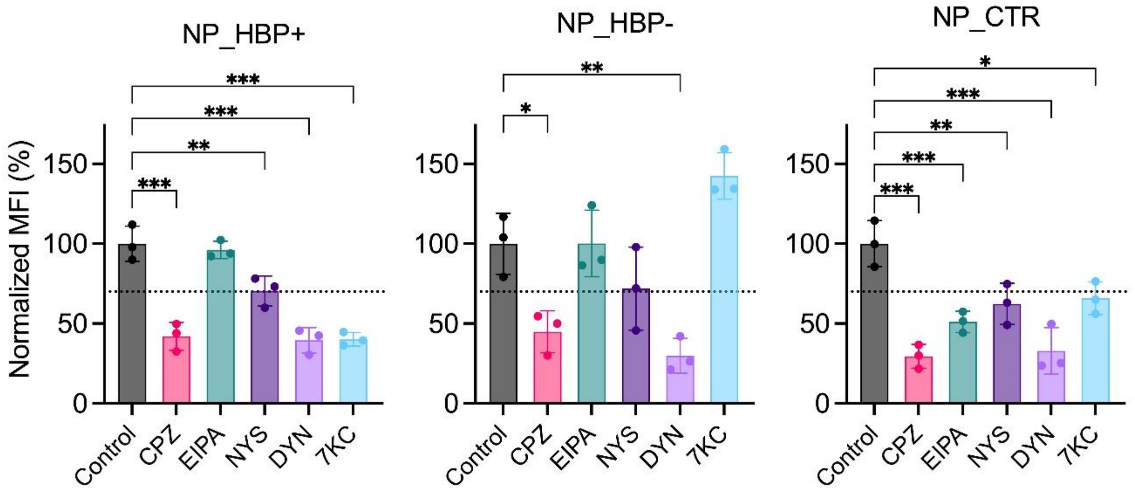

Suppl. Figure S2. Mechanisms of NP uptake in CHO-K1 cells. Pharmacological inhibitors targeting major endocytic pathways were used to assess their effects on NP uptake. The inhibited pathways included clathrin-mediated endocytosis (CME), macropinocytosis, clathrin-independent lipid raft/cholesterol-dependent pathways, and the CLIC/GEEC pathway.[1-3] The selected inhibitors included chlorpromazine (CPZ) for CME, 5-(N-ethyl-N-isopropyl)amiloride (EIPA) for macropinocytosis, nystatin (NYS) for lipid raft/cholesterol-dependent pathways, dynasore (DYN) for dynamin-dependent uptake, and 7-ketocholesterol (7KC) for CLIC/GEEC pathway. The specificity of 7KC in CHO-K1 cells was confirmed using a control marker (**Suppl. Figure S3**), while the specificity of the other inhibitors was established in our previous report.[4] Cells were pretreated with transport inhibitors for 30 min before NP exposure (50 µg mL^{-1}, 4 h, 37°C, serum-free medium). NP uptake was quantified by flow cytometry. Data are presented as normalized mean ± SD of cell fluorescence intensities from three independent replicates. The dashed line marks the 70% uptake threshold, beyond which NP uptake is considered reduced. Results from the inhibition experiments revealed that NP_HBP+ utilized both the CME and CLIC/GEEC pathways, NP_HBP− predominantly relied on CME, and NP_CTR engaged all four tested pathways with a predominance of CME.

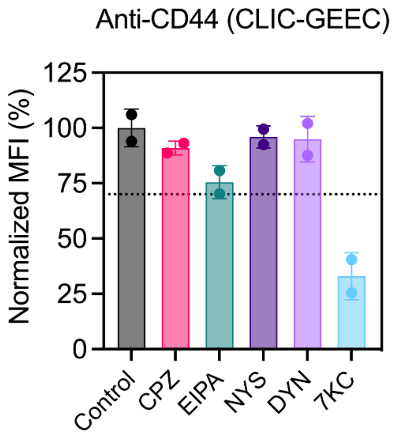

Suppl. Figure S3. Specificity of 7-ketocholesterol (7KC) as an inhibitor of the CLIC-GEEC uptake pathway in CHO-K1. Fluorescently labeled anti-CD44 was used as a control marker.[3] Cells were pre-treated with transport inhibitors for 30 min before addition of anti-CD44, and uptake was assessed by flow cytometry. Only 7KC reduced anti-CD44 uptake, while other classical endocytic inhibitors had no effect. Results are shown as normalized mean ± SD of cell fluorescence intensities from two independent replicates. The dashed line marks the 70% inhibition threshold.

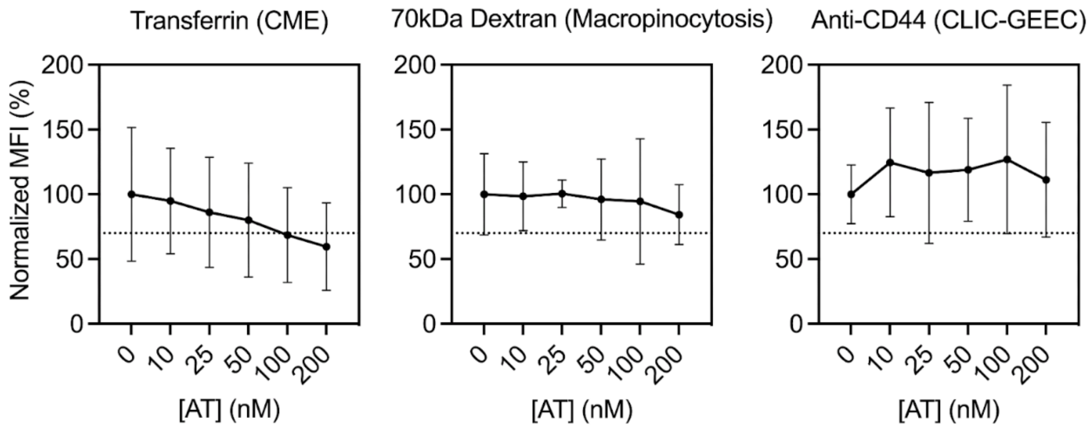

Suppl. Figure S4. Effect of AT on cell uptake of endocytic markers in CHO-K1 cells. Cells were pretreated with AT for 30 min at the indicated concentrations, then exposed to Alexa Fluor 488-labeled transferrin (25 µg mL^{-1}, CME), FITC-labeled 70 kDa dextran (250 µg mL^{-1}, macropinocytosis), or FITC-labeled anti-CD44 (100 µg mL^{-1}, CLIC/GEEC pathway). Uptake was assessed by flow cytometry. The dashed line marks the 70% inhibition threshold. Of note, the modest reduction in transferrin uptake by AT may reflect, in part, disruption of weak HSPG–transferrin interactions.[5] Data represent the normalized mean ± MFI of cell fluorescence intensities from three independent experiments.

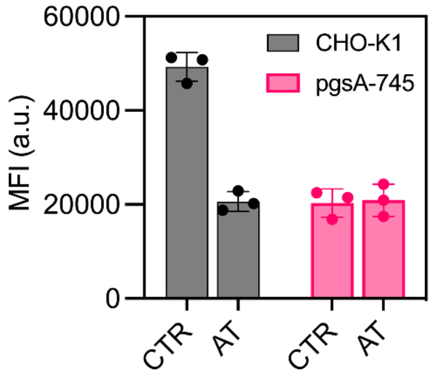

Suppl. Figure S5. Effect of antithrombin (AT) on NP_HBP+ surface adhesion. Cells were pretreated with AT (200 nM) for 30 min at 4°C, then exposed to NP_HBP+ (50 µg mL⁻¹, 4 h, 4°C, serum-free medium), followed by flow cytometry analysis. Control cells (CTR) were not treated with AT. In the presence of AT, NP surface adhesion in CHO-K1 cells decreased to the same level as in pgsA-745 cells, confirming that AT effectively blocked NP-glycocalyx interactions in CHO-K1 cells. Bars represent the mean ± SD of cell fluorescence intensities from three independent measurements.

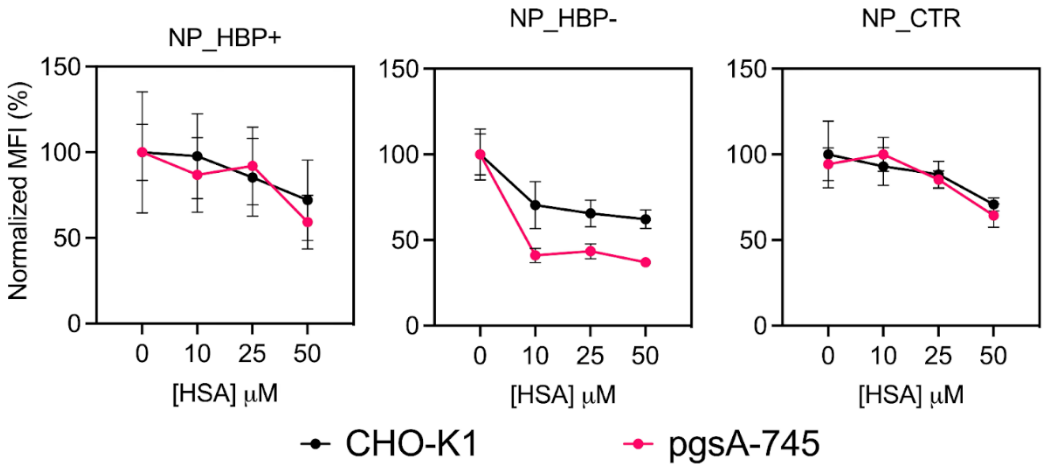

Suppl. Figure S6. Effect of HSA on NP uptake in CHO-K1 and pgsA-745 cells. Cells were pretreated with excess HSA at the indicated concentrations for 30 min. Subsequently, cells were exposed to NPs (50 µg mL⁻¹, 4 h, 37°C, serum-free medium) and uptake was quantified by flow cytometry. Data represent the normalized mean ± SD of cell fluorescence intensities from two independent measurements.

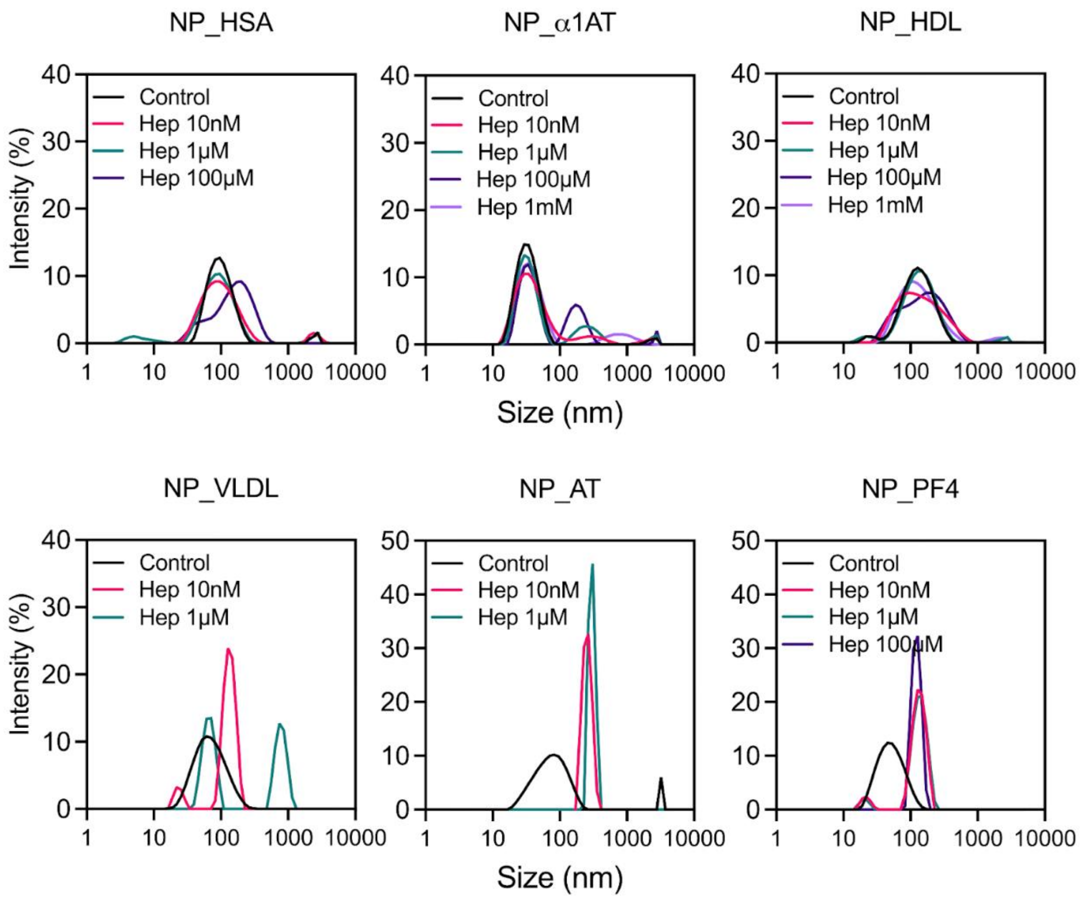

Suppl. Figure S7. DLS analysis of NP–heparin interactions via heparin-mediated NP aggregation. NPs coated with model proteins (50 µg mL^{-1}) were incubated with increasing heparin concentrations in PBS prior to DLS measurement. NPs coated with non-HBPs (NP_HSA, NP_α1AT, NP_HDL) exhibited minimal aggregation, as indicated by largely unchanged size distributions even at the highest heparin concentrations. For NP_HSA, a shift in mean size was observed only at 100 µM heparin, while NP_α1AT showed some aggregation starting at 1 µM, although the dominant signal remained at the original peak. In contrast, NPs coated with HBPs (NP_VLDL, NP_AT, NP_PF4) displayed a consistent shift of the entire size distribution peak toward larger diameters beginning at 10 nM heparin, indicative of heparin-mediated aggregation. The relatively small aggregate size for NP_PF4 likely results from efficient heparin coating of the NP surface preventing extensive NP bridging and the formation of larger aggregates.

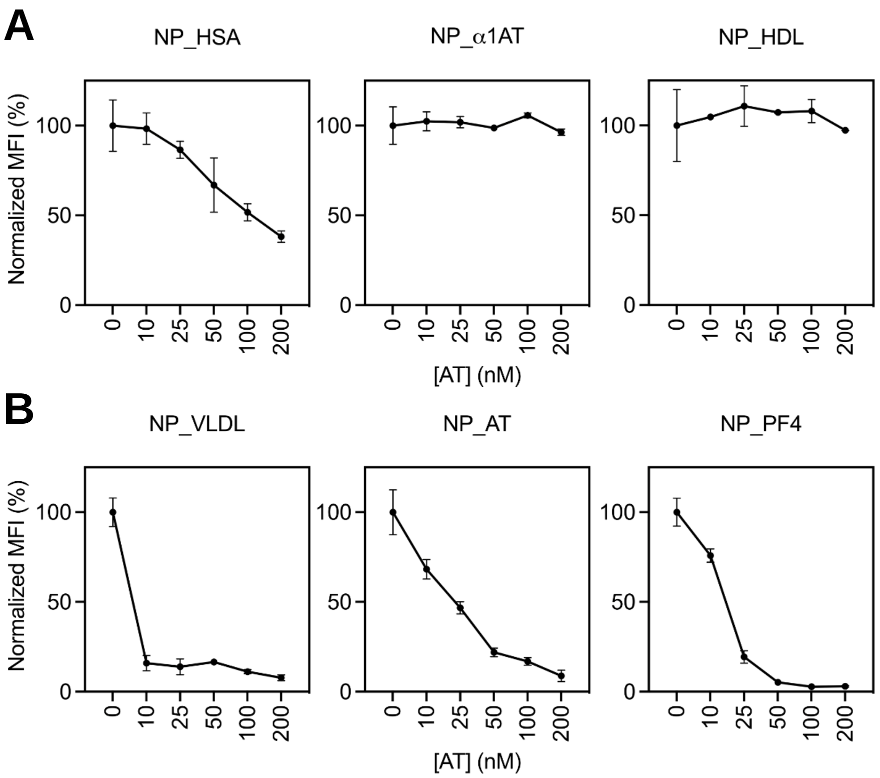

Suppl. Figure S8. Uptake of NPs coated with model coronas in CHO-K1 cells pretreated with antithrombin (AT). (A) Uptake of NPs with coronas containing non-HBPs. (B) Uptake of NPs with coronas containing HBPs. Cells were exposed to NPs (50 µg mL^{-1}, 4 h, 37°C, serum-free medium) and uptake was quantified by flow cytometry. Uptake of HBP-containing NPs decreased progressively with increasing AT concentrations. At 200 nM AT, NP_AT and NP_VLDL showed ~15-fold reductions in uptake, while NP_PF4 uptake decreased by a striking ~30-fold. In contrast, AT had no effect on the uptake of NP_α1AT and NP_HDL, and only moderately reduced NP_HSA uptake. The gradual decline in NP_HSA uptake suggests a weak contribution of HSA–glycocalyx interactions to NP internalization. This aligns with our previous findings showing that bovine serum albumin (BSA) interacts weakly with heparin and that NP_BSA uptake is partly mediated by GAG interactions.[4]

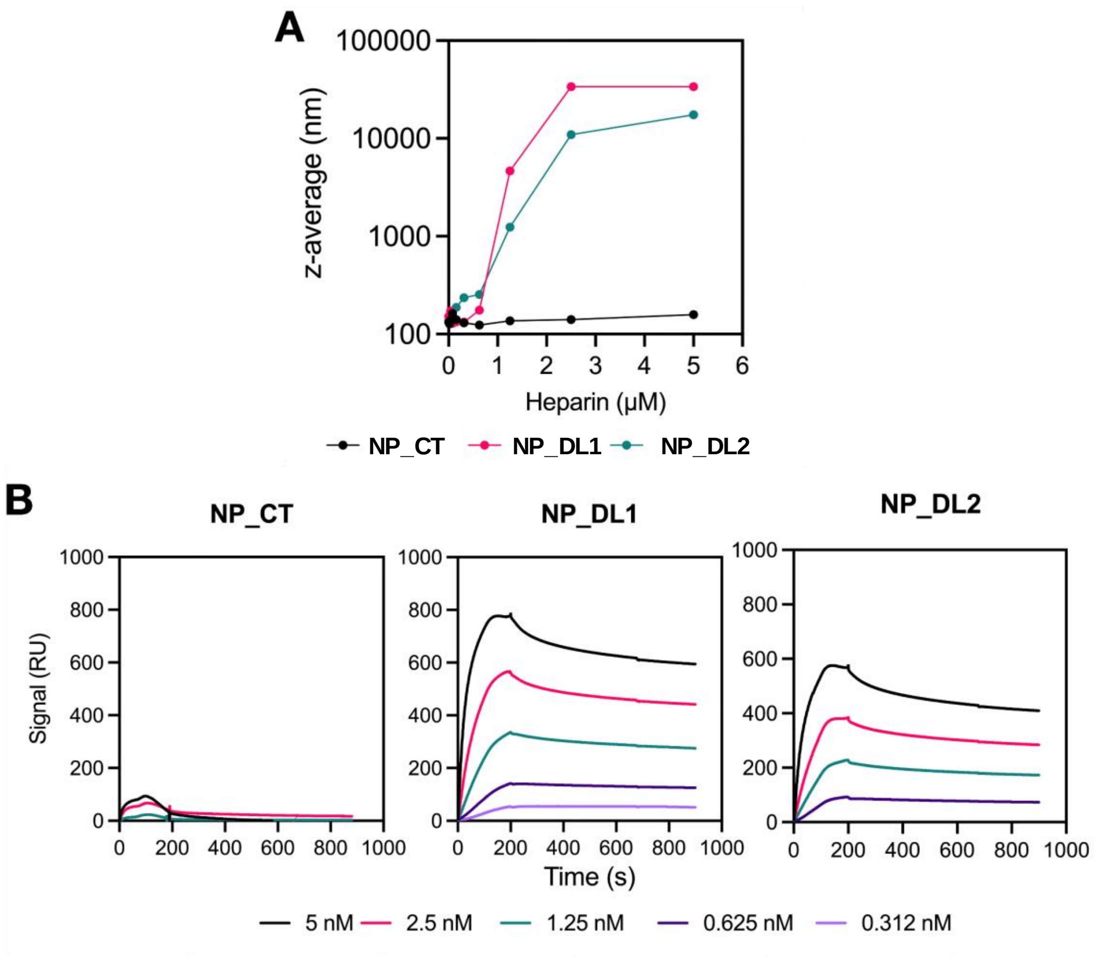

Suppl. Figure S9. Characterization of NP–heparin interactions. (A) DLS analysis. NP_CT, NP_DL1 and NP_DL2 were titrated with increasing concentrations of heparin using the instrument's automated titration module, and the corresponding z-average diameters were recorded at each titration point. Heparin-induced increases in hydrodynamic size for NP_DL1 and NP_DL2 indicated binding of heparin to their respective corona proteins. (B) SPR analysis. Binding traces revealed interactions of heparin with NP_DL1 and NP_DL2, but no significant interactions with NP_CT. Quantitative analysis with a continuous surface-site distribution model was not performed due to the poor quality of fitting.

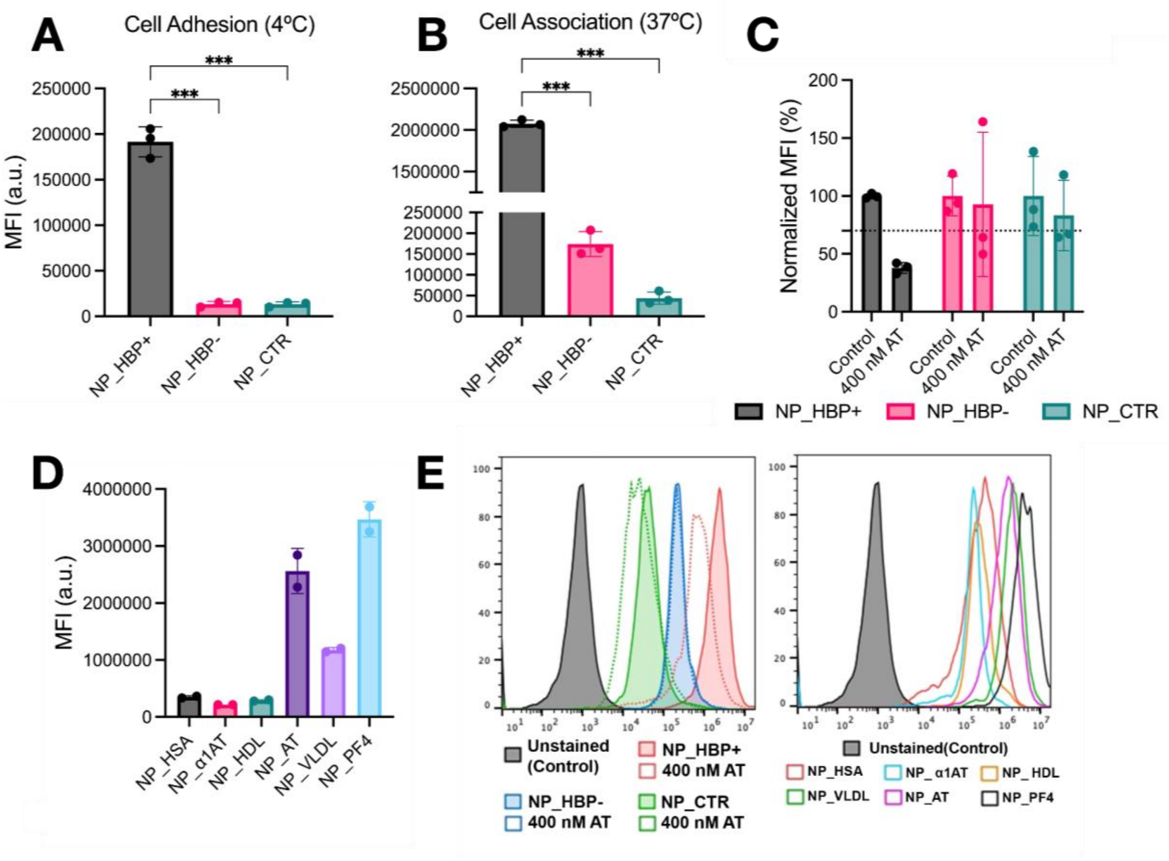

Suppl. Figure S10. NP uptake in MDA-MB-231 triple-negative breast cancer cells and the role of the glycocalyx in uptake. (A) Cell-surface adhesion of NP_HBP+, NP_HBP−, and NP_CTR evaluated at 4°C. (B) Uptake of NP_HBP+, NP_HBP−, and NP_CTR at 37°C. (C) Same as (B) after pretreatment with antithrombin (400 nm). The dashed line marks the 70% uptake threshold, beyond which NP uptake is considered reduced. (D) Uptake of model NPs with tailored coronas containing HSA mixed with HBPs or non-HBPs. (E) Sample histograms showing fluorescence intensity distributions used to derive MFI values of NP uptake. Cells were exposed to NPs (50 µg mL^{-1}, 4 h, serum-free medium) and uptake was quantified by flow cytometry. Data represent mean (or normalized mean) ± SD of cell fluorescence intensities from two or three independent experiments.

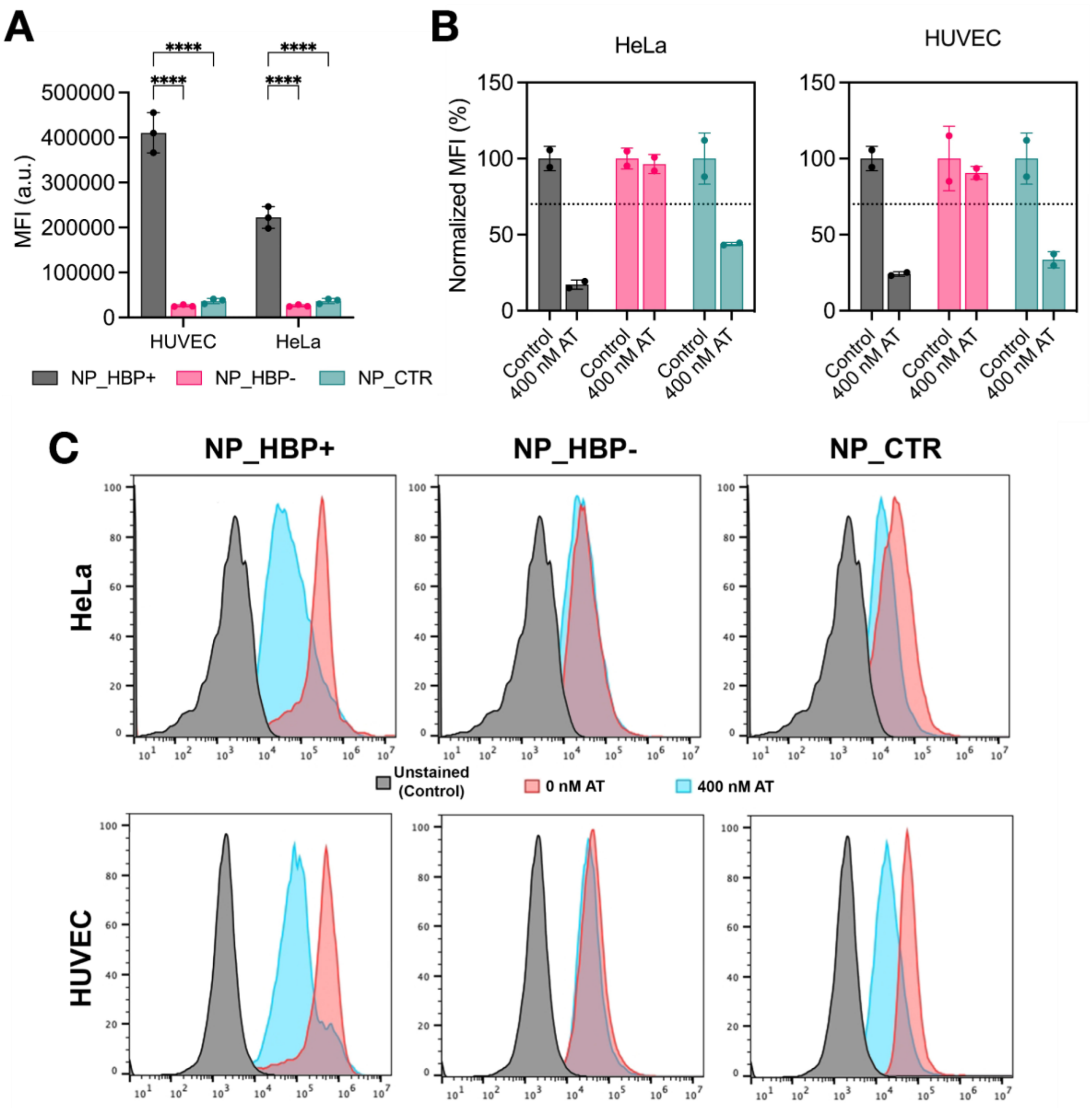

Suppl. Figure S11. NP uptake in HUVEC and HeLa cells and the role of the glycocalyx. (A) Uptake of NP_HBP+, NP_HBP−, and NP_CTR. (B) Same as (A) after pretreatment with antithrombin (400 nM). The dashed line marks the 70% uptake threshold, beyond which NP uptake is considered reduced. (C) Sample histograms showing fluorescence intensity distributions used to derive MFI values of NP uptake. Cells were exposed to NPs (50 µg mL^{-1}, 2 or 4 h (HeLa or HUVEC), 37°C, serum-free medium), and uptake was quantified by flow cytometry. Bars represent the mean (or normalized mean) ± SD of cell fluorescence intensities from two or three independent measurements.

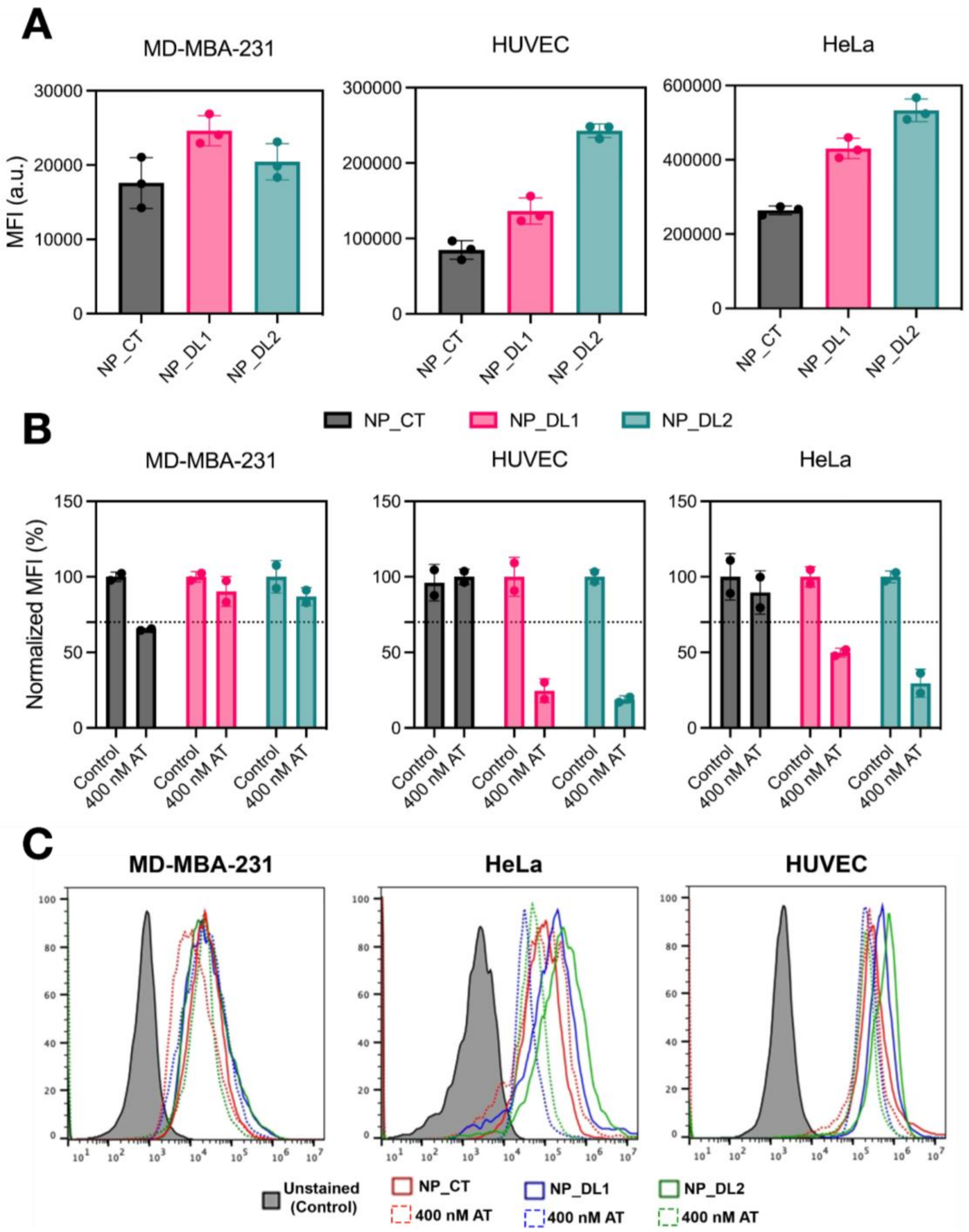

Suppl. Figure S12. Uptake of NPs coated with physiologically derived protein coronas in MDA-MB-231, HUVEC, and HeLa cells and the role of the glycocalyx. (A) Uptake of NP_CT, NP_DL1, and NP_DL2 in MDA-MB-231, HUVEC, and HeLa cells. (B) Same as (A) after pretreatment with antithrombin (400 nm). The dashed line marks the 70% uptake threshold, beyond which NP uptake is considered reduced. (C) Sample histograms showing fluorescence intensity distributions used to derive MFI values of NP uptake. Cells were exposed to NPs (50 µg mL^{-1}, 2 or 4 h, 37°C, serum-free medium), and uptake was quantified by flow cytometry. Bars represent the mean (or normalized mean) ± SD of cell fluorescence intensities from two or three independent measurements.

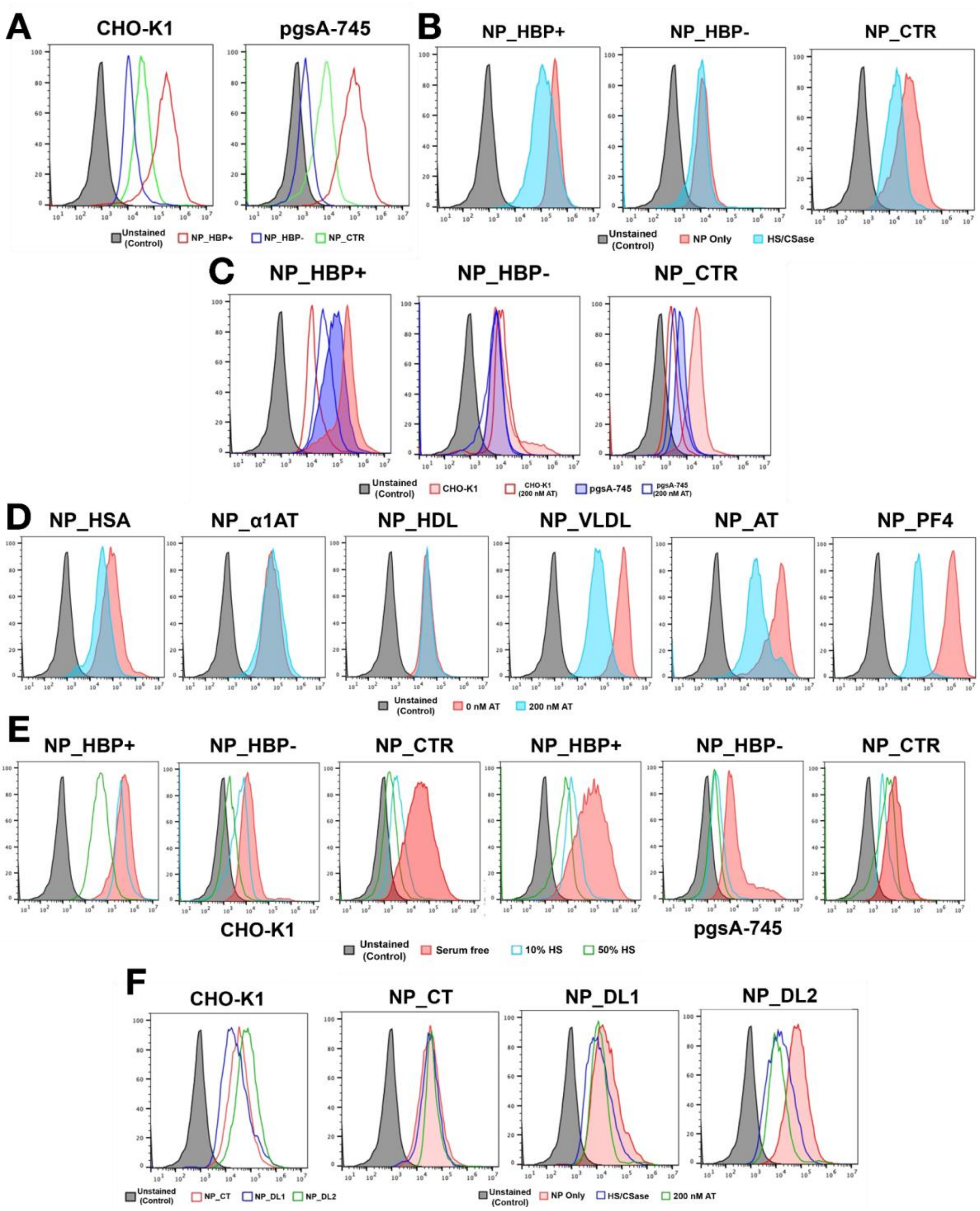

Suppl. Figure S13. Sample flow cytometry histograms showing fluorescence intensity distributions used to derive MFI values of NP uptake. The displayed histograms correspond to the following figures in the main text: (A) Figure 5. (B) Figure 6B (NP uptake by pristine and enzyme-treated CHO-K1 cells). (C) Figure 6C (NP uptake by CHO-K1 and pgsA-745 cells in the absence or presence of 200 nM AT). (D) Figure 7. (E) Figure 8. (F) Figure 9 (NP uptake by pristine and enzyme-treated CHO-K1 cells, and CHO-K1 cells with or without 200 nM AT).

Suppl. Table S1. Proteins identified in the HBP+, HBP−, and CTR biofluids, along with their RPA (%) values.

Index	Gene	Description	HBP+	HBP-	CTR
**1**	**ANT3**	Antithrombin-III	88.0600	0.0633	0.1749
**2**	**CO8A**	Complement component C8 alpha chain	7.5363	0.0123	0.0213
**3**	**ALBU**	Albumin	1.1063	72.2376	70.4257
**4**	**PLF4**	Platelet factor 4	0.5144	0.0006	0.0009
**5**	**FA8**	Coagulation Factor XIII alpha	0.3133	0.1262	0.0922
**6**	**APOE**	Apolipoprotein E	0.2709	0.0589	0.0589
**7**	**IGG1**	Immunoglobulin gamma-1 heavy chain	0.1940	3.1004	2.8501
**8**	**AUP1**	Lipid droplet-regulating VLDL assembly factor AUP1	0.1621	0.0344	0.0052
**9**	**IGK**	Immunoglobulin kappa light chain	0.1295	1.1707	1.3604
**10**	**IGKC**	Immunoglobulin kappa constant	0.1293	1.1682	1.3575
**11**	**HPT**	Haptoglobin	0.1023	2.4850	2.7317
**12**	**FIBG**	Fibrinogen gamma chain	0.0945	0.6996	0.8952
**13**	**IGHG2**	Immunoglobulin heavy constant gamma 2	0.0897	1.8804	1.7067
**14**	**IGLC2**	Immunoglobulin lambda constant 2	0.0888	2.0461	2.2128
**15**	**FIBB**	Fibrinogen beta chain	0.0864	0.7232	1.1140
**16**	**KNG1**	Kininogen-1	0.0655	0.1063	0.1288
**17**	**TRFE**	Serotransferrin	0.0579	2.2330	1.9739
**18**	**HRG**	Histidine-rich glycoprotein	0.0553	0.0490	0.0520
**19**	**IGHG4**	Immunoglobulin heavy constant gamma 4	0.0474	1.4478	1.2833
**20**	**CO3**	Complement C3	0.0457	0.2428	0.2317
**21**	**IGHM**	Immunoglobulin heavy constant mu	0.0418	0.0644	0.0791
**22**	**IGHA1**	Immunoglobulin heavy constant alpha 1	0.0415	0.8846	0.9259
**23**	**APOB**	Apolipoprotein B-100	0.0383	0.0538	0.0722
**24**	**TRFL**	Lactotransferrin	0.0380	0.0006	0.0005
**25**	**ACTB**	Actin cytoplasmic 1	0.0377	0.0181	0.0198
**26**	**CXCL7**	Platelet basic protein	0.0351	0.0022	0.0018
**27**	**ANGI**	Angiogenin	0.0343	0.1597	0.1530
**28**	**APOH**	Beta-2-glycoprotein 1	0.0333	0.3069	0.2730
**29**	**IGHG3**	Immunoglobulin heavy constant gamma 3	0.0279	0.7167	0.6492

**30**	**FIBA**	Fibrinogen alpha chain	0.0255	0.6728	0.9270
**31**	**FINC**	Fibronectin	0.0236	0.0655	0.0372
**32**	**IBP3**	Insulin-like growth factor-binding protein 3	0.0209	0.0012	0.0016
**33**	**KV320**	Immunoglobulin kappa variable 3-20	0.0199	0.1540	0.1804
**34**	**A1AT**	Alpha-1-antitrypsin	0.0189	0.5433	0.7824
**35**	**AMBP**	Protein AMBP	0.0181	0.0321	0.0426
**36**	**IGLL5**	Immunoglobulin lambda-like polypeptide 5	0.0167	0.4538	0.4971
**37**	**F111B**	Serine protease FAM111B	0.0141	0.0036	0.0055
**38**	**HEMO**	Hemopexin	0.0140	0.6067	0.7840
**39**	**FA9**	Coagulation factor IX	0.0133	0.0007	0.0010
**40**	**CFAD**	Complement factor D	0.0118	0.0006	0.0005
**41**	**RARR2**	Retinoic acid receptor responder protein 2	0.0111	0.0010	0.0011
**42**	**A1AG1**	Alpha-1-acid glycoprotein 1	0.0105	0.2875	0.2884
**43**	**TSP1**	Thrombospondin-1	0.0097	0.0063	0.0059
**44**	**VTNC**	Vitronectin	0.0095	0.0715	0.1174
**45**	**APOA1**	Apolipoprotein A-I	0.0092	0.6524	0.5110
**46**	**HABP2**	Hyaluronan-binding protein 2	0.0091	0.1940	0.0684
**47**	**VTDB**	Vitamin D-binding protein	0.0085	0.1335	0.2416
**48**	**PRG4**	Proteoglycan 4	0.0085	0.0333	0.0522
**49**	**TBA3C**	Tubulin alpha-3C chain	0.0083	0.0002	0.0002
**50**	**CAVN2**	Caveolae-associated protein 2	0.0082	0.0001	0.0001
**51**	**APOA2**	Apolipoprotein A-II	0.0081	0.0916	0.1858
**52**	**H2B1K**	Histone H2B type 1-K	0.0076	0.0014	0.0015
**53**	**VIME**	Vimentin	0.0075	0.0077	0.0058
**54**	**CERU**	Ceruloplasmin	0.0072	0.1663	0.1507
**55**	**KLKB1**	Plasma kallikrein	0.0072	0.0054	0.0087
**56**	**SODE**	Extracellular superoxide dismutase [Cu-Zn]	0.0068	0.0000	0.0001
**57**	**C4BPA**	C4b-binding protein alpha chain	0.0068	0.0478	0.0654
**58**	**ITIH2**	Inter-alpha-trypsin inhibitor heavy chain H2	0.0065	0.1043	0.0987
**59**	**KVD11**	Immunoglobulin kappa variable 3D-11	0.0062	0.1258	0.0871
**60**	**KV228**	Immunoglobulin kappa variable 2-28	0.0056	0.0512	0.0678
**61**	**IGA2**	Immunoglobulin alpha-2 heavy chain	0.0055	0.2653	0.2657

**62**	**CLUS**	Clusterin	0.0053	0.0506	0.0762
**63**	**KV401**	Immunoglobulin kappa variable 4-1	0.0053	0.0493	0.0814
**64**	**TFPI1**	Tissue factor pathway inhibitor	0.0051	0.0000	0.0001
**65**	**TTHY**	Transthyretin	0.0050	0.0153	0.0163
**66**	**PLEK**	Pleckstrin	0.0049	0.0003	0.0002
**67**	**HV307**	Immunoglobulin heavy variable 3-7	0.0048	0.0355	0.0702
**68**	**CFAB**	Complement factor B	0.0044	0.0889	0.1186
**69**	**FETUA**	Alpha-2-HS-glycoprotein	0.0044	0.2038	0.2109
**70**	**LV325**	Immunoglobulin lambda variable 3-25	0.0042	0.0208	0.0299
**71**	**AACT**	Alpha-1-antichymotrypsin	0.0039	0.1032	0.1226
**72**	**FA5**	Coagulation factor V	0.0039	0.0002	0.0004
**73**	**CO4A**	Complement C4-A	0.0038	0.0859	0.1006
**74**	**EF1A1**	Elongation factor 1-alpha 1	0.0037	0.0014	0.0007
**75**	**HV315**	Immunoglobulin heavy variable 3-15	0.0037	0.0798	0.0455
**76**	**A2MG**	Alpha-2-macroglobulin	0.0037	0.5664	0.6921
**77**	**A2AP**	Alpha-2-antiplasmin	0.0035	0.0278	0.0287
**78**	**IGJ**	Immunoglobulin J chain	0.0030	0.0175	0.0279
**79**	**PLMN**	Plasminogen	0.0029	0.1007	0.0913
**80**	**HV366**	Immunoglobulin heavy variable 3-66	0.0027	0.0615	0.0828
**81**	**A1BG**	Alpha-1B-glycoprotein	0.0027	0.0847	0.0820
**82**	**GELS**	Gelsolin	0.0027	0.0337	0.0302
**83**	**PON1**	Serum paraoxonase/arylesterase 1	0.0026	0.0195	0.0363
**84**	**CD5L**	CD5 antigen-like	0.0024	0.0120	0.0160
**85**	**IBP5**	Insulin-like growth factor-binding protein 5	0.0024	0.0000	0.0001
**86**	**HGFL**	Hepatocyte growth factor-like protein	0.0023	0.0003	0.0001
**87**	**HV461**	Immunoglobulin heavy variable 4-61	0.0022	0.0555	0.0349
**88**	**ENOA**	Alpha-enolase	0.0021	0.0001	0.0002
**89**	**CO4B**	Complement C4-B	0.0020	0.0446	0.0521
**90**	**THRB**	Prothrombin	0.0019	0.0852	0.0727
**91**	**LBP**	Lipopolysaccharide-binding protein	0.0018	0.0001	0.0001
**92**	**COF1**	Cofilin-1	0.0018	0.0042	0.0011
**93**	**C1S**	Complement C1s subcomponent	0.0016	0.0080	0.0072

**94**	**IC1**	Plasma protease C1 inhibitor	0.0016	0.0341	0.0350
**95**	**H4**	Histone H4	0.0016	0.0007	0.0017
**96**	**TETN**	Tetranectin	0.0016	0.0035	0.0029
**97**	**HV64D**	Immunoglobulin heavy variable 3-64D	0.0015	0.0133	0.0231
**98**	**C1QB**	Complement C1q subcomponent subunit B	0.0015	0.0093	0.0121
**99**	**HV374**	Immunoglobulin heavy variable 3-74	0.0015	0.0186	0.0317
**100**	**FA12**	Coagulation factor XII	0.0015	0.0080	0.0054
**101**	**ITIH4**	Inter-alpha-trypsin inhibitor heavy chain H4	0.0015	0.0359	0.0462
**102**	**CFAH**	Complement factor H	0.0014	0.1079	0.0875
**103**	**EF2**	Elongation factor 2	0.0014	0.0018	0.0041
**104**	**HPTR**	Haptoglobin-related protein	0.0014	0.0268	0.0282
**105**	**KVD30**	Immunoglobulin kappa variable 2D-30	0.0013	0.0236	0.0189
**106**	**CAP7**	Azurocidin	0.0013	0.0000	0.0001
**107**	**CFAI**	Complement factor I	0.0012	0.0085	0.0098
**108**	**C1QC**	Complement C1q subcomponent subunit C	0.0012	0.0077	0.0121
**109**	**LV319**	Immunoglobulin lambda variable 3-19	0.0011	0.0196	0.0370
**110**	**PI42A**	Phosphatidylinositol 5-phosphate 4-kinase type-2 alpha	0.0010	0.0000	0.0000
**111**	**ITIH1**	Inter-alpha-trypsin inhibitor heavy chain H1	0.0010	0.0693	0.0741
**112**	**FA11**	Coagulation factor XI	0.0010	0.0000	0.0001
**113**	**CO9**	Complement component C9	0.0010	0.0112	0.0101
**114**	**CO8G**	Complement component C8 gamma chain	0.0009	0.0070	0.0137
**115**	**HBA**	Hemoglobin subunit alpha	0.0009	0.0054	0.0043
**116**	**VWF**	von Willebrand factor	0.0009	0.0003	0.0003
**117**	**HV348**	Immunoglobulin heavy variable 3-48	0.0009	0.0214	0.0352
**118**	**APOD**	Apolipoprotein D	0.0009	0.0128	0.0302
**119**	**MYH9**	Myosin-9	0.0009		
**120**	**LV321**	Immunoglobulin lambda variable 3-21	0.0008	0.0494	0.0437
**121**	**CO5**	Complement C5	0.0008	0.0064	0.0108
**122**	**SAMP**	Serum amyloid P-component	0.0008	0.0365	0.0342
**123**	**APOC3**	Apolipoprotein C-III	0.0007	0.0326	0.0470
**124**	**HV372**	Immunoglobulin heavy variable 3-72	0.0007	0.0157	0.0200

**125**	**PGRP2**	N-acetylmuramoyl-L-alanine amidase	0.0007	0.0145	0.0134
**126**	**HV43D**	Immunoglobulin heavy variable 3-43D	0.0006	0.0083	0.0113
**127**	**C1R**	Complement C1r subcomponent	0.0006	0.0165	0.0091
**128**	**HV335**	Probable non-functional immunoglobulin heavy variable 3-35	0.0006	0.0101	0.0177
**129**	**C1QA**	Complement C1q subcomponent subunit A	0.0006	0.0032	0.0063
**130**	**HV601**	Immunoglobulin heavy variable 6-1	0.0006	0.0158	0.0109
**131**	**FHR1**	Complement factor H-related protein 1	0.0006	0.0039	0.0049
**132**	**HEP2**	Heparin cofactor 2	0.0006	0.0259	0.0231
**133**	**KV109**	Immunoglobulin kappa variable 1-9	0.0005	0.0076	0.0139
**134**	**LV743**	Immunoglobulin lambda variable 7-46	0.0005	0.0089	0.0152
**135**	**FBLN1**	Fibulin-1	0.0004	0.0041	0.0050
**136**	**HBB**	Hemoglobin subunit beta	0.0004	0.0070	0.0119
**137**	**KVD15**	Immunoglobulin kappa variable 3D-15	0.0004	0.0279	0.0254
**138**	**KVD13**	Immunoglobulin kappa variable 1D-13	0.0004	0.0205	0.0192
**139**	**HV551**	Immunoglobulin heavy variable 5-51	0.0004	0.0584	0.0880
**140**	**ECM1**	Extracellular matrix protein 1	0.0004	0.0018	0.0010
**141**	**PROS**	Vitamin K-dependent protein S	0.0004	0.0109	0.0094
**142**	**HV69D**	Immunoglobulin heavy variable 1-69D	0.0003	0.0122	0.0128
**143**	**ANGT**	Angiotensinogen	0.0003	0.0122	0.0147
**144**	**ATRN**	Attractin	0.0003	0.0022	0.0033
**145**	**KV240**	Immunoglobulin kappa variable 2-40	0.0003	0.0017	0.0026
**146**	**LV151**	Immunoglobulin lambda variable 1-51	0.0003	0.0034	0.0024
**147**	**KVD33**	Immunoglobulin kappa variable 1D-33	0.0003	0.0155	0.0197
**148**	**PEDF**	Pigment epithelium-derived factor	0.0003	0.0051	0.0070
**149**	**APOA4**	Apolipoprotein A-IV	0.0003	0.0531	0.0864
**150**	**AFAM**	Afamin	0.0003	0.0327	0.0492
**151**	**APOM**	Apolipoprotein M	0.0003	0.0090	0.0092
**152**	**KV139**	Immunoglobulin kappa variable 1-39	0.0003	0.0095	0.0114
**153**	**LV861**	Immunoglobulin lambda variable 8-61	0.0003	0.0024	0.0033
**154**	**ZA2G**	Zinc-alpha-2-glycoprotein	0.0003	0.0199	0.0318

**155**	**CBG**	Corticosteroid-binding globulin	0.0002	0.0085	0.0055
**156**	**CO8B**	Complement component C8 beta chain	0.0002	0.0063	0.0089
**157**	**LV147**	Immunoglobulin lambda variable 1-47	0.0002	0.0196	0.0184
**158**	**HV313**	Immunoglobulin heavy variable 3-13	0.0002	0.0037	0.0070
**159**	**KV224**	Immunoglobulin kappa variable 2-24	0.0002	0.0061	0.0071
**160**	**ALS**	Insulin-like growth factor-binding protein complex acid labile subunit	0.0002	0.0056	0.0053
**161**	**A1AG2**	Alpha-1-acid glycoprotein 2	0.0002	0.0575	0.0832
**162**	**KVD29**	Immunoglobulin kappa variable 2D-29	0.0002	0.0012	0.0038
**163**	**CBPB2**	Carboxypeptidase B2	0.0001	0.0018	0.0022
**164**	**CO6**	Complement component C6	0.0001	0.0042	0.0046
**165**	**HV364**	Immunoglobulin heavy variable 3-64	0.0001	0.0070	0.0066
**166**	**LG3BP**	Galectin-3-binding protein	0.0001	0.0028	0.0028
**167**	**IGD**	Immunoglobulin delta heavy chain	0.0001	0.0026	0.0028
**168**	**F13B**	Coagulation factor XIII B chain	0.0001	0.0011	0.0023
**169**	**SAA4**	Serum amyloid A-4 protein	0.0001	0.0146	0.0216
**170**	**HV373**	Immunoglobulin heavy variable 3-73	0.0001	0.0047	0.0035
**171**	**CO2**	Complement C2	0.0001	0.0034	0.0030
**172**	**A2GL**	Leucine-rich alpha-2-glycoprotein	0.0001	0.0054	0.0057
**173**	**CPN2**	Carboxypeptidase N subunit 2		0.0025	0.0037
**174**	**AHNK**	Neuroblast differentiation-associated protein AHNAK		0.0071	0.0037
**175**	**FCN3**	Ficolin-3		0.0056	0.0078
**176**	**CBPN**	Carboxypeptidase N catalytic chain		0.0036	0.0032
**177**	**LUM**	Lumican		0.0041	0.0047
**178**	**FHR2**	Complement factor H-related protein 2		0.0007	0.0008
**179**	**THBG**	Thyroxine-binding globulin		0.0046	0.0071
**180**	**ITIH3**	Inter-alpha-trypsin inhibitor heavy chain H3		0.0014	0.0022
**181**	**CO7**	Complement component C7		0.0069	0.0082
**182**	**APOL1**	Apolipoprotein L1		0.0047	0.0079
**183**	**FA10**	Coagulation factor X		0.0011	0.0011
**184**	**APOC2**	Apolipoprotein C-II		0.0018	0.0120
**185**	**RET4**	Retinol-binding protein 4		0.0171	0.0204

**186**	**KVD21**	Immunoglobulin kappa variable 6D-21		0.0018	0.0027
**187**	**KV117**	Immunoglobulin kappa variable 1-17		0.0022	0.0025
**188**	**PZP**	Pregnancy zone protein		0.0006	0.0013
**189**	**HV118**	Immunoglobulin heavy variable 1-18		0.0009	0.0009
**190**	**KV105**	Immunoglobulin kappa variable 1-5		0.0034	0.0012
**191**	**SAA1**	Serum amyloid A-1 protein		0.0013	0.0020
**192**	**KAIN**	Kallistatin		0.0031	0.0053
**193**	**KVD16**	Immunoglobulin kappa variable 1D-16		0.0006	0.0007
**194**	**PHLD**	Phosphatidylinositol-glycan-specific phospholipase D		0.0014	0.0009
**195**	**KV116**	Immunoglobulin kappa variable 1-16		0.0007	0.0003
**196**	**HV383**	Probable non-functional immunoglobulin heavy variable 3-38-3		0.0006	0.0008
**197**	**KV137**	Probable non-functional immunoglobulinn kappa variable 1-37		0.0029	0.0024
**198**	**DNHD1**	Dynein heavy chain domain-containing protein 1		0.0000	0.0005
**199**	**EIF3G**	Eukaryotic translation initiation factor 3 subunit G		0.0195	0.0170
**200**	**R1AB**	Replicase polyprotein 1ab		0.0004	0.0006
**201**	**HV320**	Immunoglobulin heavy variable 3-20		0.0008	0.0001
**202**	**FHR3**	Complement factor H-related protein 3		0.0008	0.0001

Suppl. Table S2. NP characterization in terms of hydrodynamic diameter (HD), polydispersity index (PDI), zeta potential (ZP), and adsorbed protein mass determined by the micro-BCA assay, converted to the estimated number of adsorbed proteins per NP (n) assuming an average protein molecular weight of 60 kDa.

Nanoparticle	n	HD (nm)	PDI	ZP (mV)
Bare	-	49 ± 1	0.06	-33 ± 7
NP_HBP+	81	81 ± 16	0.25	-26 ± 1
NP_HBP−	67	87 ± 12	0.22	-25 ± 2
NP_CTR	149	86 ± 9	0.16	-27 ± 1

Suppl. Table S3. Proteins identified in the coronas of NP_HBP+, HP_HBP−, and NP_CTR, along with their RPA (%) values. Only proteins with RPA > 0.1% in at least one corona are included.

Gene	Description	NP_HBP+	NP_HBP-	NP_CTR
**APOE**	Apolipoprotein E	79.59	0.76	0.54
**PLF4**	Platelet factor 4	7.92	0.01	0.04
**ANT3**	Antithrombin-III	2.50	0.00	0.01
**APOA1**	Apolipoprotein A-I	1.34	67.87	83.28
**ALBU**	Albumin	0.50	1.82	1.95
**KNG1**	Kininogen-1	0.50	0.81	0.20
**HRG**	Histidine-rich glycoprotein	0.48	1.19	0.51
**FIBG**	Fibrinogen gamma chain	0.40	0.83	0.48
**FIBB**	Fibrinogen beta chain	0.34	0.58	0.39
**ITB6**	Integrin beta-6	0.32	0.08	0.05
**APOA2**	Apolipoprotein A-II	0.28	8.29	3.98
**APOB**	Apolipoprotein B-100	0.28	1.51	0.42
**FIBA**	Fibrinogen alpha chain	0.28	0.62	0.55
**VTNC**	Vitronectin	0.24	1.12	0.20
**FINC**	Fibronectin	0.24	0.02	0.01

**CCD40**	Coiled-coil domain-containing protein 40	0.21	0.01	0.01
**LBP**	Lipopolysaccharide-binding protein	0.17	0.04	0.01
**IGG1**	Immunoglobulin gamma-1 heavy chain	0.15	0.26	0.24
**GWL**	Serine/threonine-protein kinase greatwall	0.15	0.00	0.00
**CO3**	Complement C3	0.14	0.55	0.38
**A1AT**	Alpha-1-antitrypsin	0.13	2.53	1.53
**HABP2**	Hyaluronan-binding protein 2	0.12	0.18	0.11
**H14**	Histone H1.4	0.12	0.16	0.10
**RARR2**	Retinoic acid receptor responder protein 2	0.12	0.00	0.00
**CLUS**	Clusterin	0.11	0.34	0.23
**IGKC**	Immunoglobulin kappa constant	0.11	0.45	0.36
**ANGI**	Angiogenin	0.11	0.00	0.00
**APOC2**	Apolipoprotein C-II	0.10	0.33	0.15
**CAVN2**	Caveolae-associated protein 2	0.10	0.00	0.00
**APOC3**	Apolipoprotein C-III	0.09	2.61	0.47
**IGLC2**	Immunoglobulin lambda constant 2	0.09	0.19	0.15
**APOC1**	Apolipoprotein C-I	0.08	0.58	0.21
**LITD1**	LINE-1 type transposase domain-containing protein 1	0.07	0.15	0.16
**IGK**	Immunoglobulin kappa light chain	0.07	0.27	0.21
**ITIH2**	Inter-alpha-trypsin inhibitor heavy chain H2	0.06	0.43	0.04
**APOD**	Apolipoprotein D	0.06	0.31	0.26
**IGL1**	Immunoglobulin lambda-1 light chain	0.06	0.12	0.07
**IGHA1**	Immunoglobulin heavy constant alpha 1	0.06	0.17	0.09
**CO1A1**	Collagen alpha-1(I) chain	0.04	0.21	0.04
**IGHM**	Immunoglobulin heavy constant mu	0.04	0.12	0.14
**IGM**	Immunoglobulin mu heavy chain	0.03	0.09	0.11
**PON1**	Serum paraoxonase/arylesterase 1	0.02	0.14	0.10

**HPT**	Haptoglobin	0.02	0.10	0.07
**CO4B**	Complement C4-B	0.02	0.31	0.12
**A2MG**	Alpha-2-macroglobulin	0.02	0.11	0.06
**PRG4**	Proteoglycan 4	0.02	0.11	0.02
**APOL1**	Apolipoprotein L1	0.02	0.18	0.14
**SEPP1**	Selenoprotein P	0.01	0.14	0.06
**CO4A**	Complement C4-A	0.01	0.14	0.06
**SAA4**	Serum amyloid A-4 protein	0.01	0.10	0.05
**APOA4**	Apolipoprotein A-IV	0.01	0.15	0.05
**FA12**	Coagulation factor XII	0.01	0.13	0.02

Suppl. Table S4. Proteins identified in the coronas of NP_CT, HP_DL1, and NP_DL2, along with their RPA (%) values. Only proteins with RPA > 0.1% in at least one corona are included.

**Gene**	**Description**	**NP_CT**	**NP_DL1**	**NP_DL2**
**APOA1**	Apolipoprotein A-I	40.12232	28.00688	18.11924
**APOA2**	Apolipoprotein A-II	17.12251	13.10787	10.38032
**ALB**	Albumin	11.66332	7.83944	14.50789
**APOE**	Apolipoprotein E	3.20876	4.79357	5.48829
**IGK**	Immunoglobulin kappa light chain	1.98953	3.76936	4.15201
**APOD**	Apolipoprotein D	1.92408	2.30785	1.88949
**IGHM**	Immunoglobulin heavy constant mu	1.45246	3.09403	2.56746
**APOC3**	Apolipoprotein C-III	1.25968	1.73799	1.60834
**IGLC1**	Immunoglobulin lambda-1 light chain	1.09590	1.86334	2.10473
**APOB**	Apolipoprotein B-100	1.09287	1.83686	5.37667
**SAA4**	Serum amyloid A-4 protein	0.96530	1.07412	1.82175
**IGHA1**	Immunoglobulin heavy constant alpha 1	0.95716	1.39952	1.21874
**APOA4**	Apolipoprotein A-IV	0.92834	0.76805	0.81035
**HRG**	Histidine-rich glycoprotein	0.78117	3.97305	1.08337

**IGLC2**	Immunoglobulin lambda constant 2	0.73329	1.32556	1.49784
**APOM**	Apolipoprotein M	0.72716	0.78931	0.97954
**SERPIN A1**	Alpha-1-antitrypsin	0.72460	0.29531	0.31696
**IGHG2**	Immunoglobulin heavy constant gamma 2	0.70872	2.17620	2.92575
**A2M**	Alpha-2-macroglobulin	0.51909	0.53564	0.48043
**PF4**	Platelet factor 4	0.50766	1.20117	1.53085
**LGALS3 BP**	Galectin-3-binding protein	0.38946	0.53517	0.45023
**LPA**	Apolipoprotein(a)	0.38746	0.74462	1.84672
**HBB**	Hemoglobin subunit beta	0.38453	0.56721	0.91729
**CLU**	Clusterin	0.36704	0.41550	0.40359
**HPR**	Haptoglobin-related protein	0.35564	0.31071	0.44014
**HP**	Haptoglobin	0.33297	0.37196	0.41781
**PON1**	Serum paraoxonase/arylesterase 1	0.32251	0.31526	0.37148
**VTN**	Vitronectin	0.30794	0.44717	0.46548
**HBA1**	Hemoglobin subunit alpha	0.30265	0.51703	0.77206
**JCHAIN**	Immunoglobulin J chain	0.29674	0.47389	0.41507
**IGG1**	Immunoglobulin gamma-1 heavy chain	0.28650	0.70508	1.17112
**ORM2**	Alpha-1-acid glycoprotein 2	0.27187	0.34640	0.28037
**APOL1**	Apolipoprotein L1	0.25277	0.23426	0.53527
**PON3**	Serum paraoxonase/lactonase 3	0.24612	0.27027	0.38389
**TF**	Transferrin	0.23762	0.17523	0.31082
**KNG1**	Kininogen-1	0.21725	0.37090	0.18964
**SELENO P**	Selenoprotein P	0.20832	0.05262	0.03696
**SAA1**	Serum amyloid A-1 protein	0.20742	0.95581	0.18552
**IGKC**	Immunoglobulin kappa constant	0.20379	0.43829	0.35555
**APOC2**	Apolipoprotein C-II	0.18560	0.53033	0.53630

**IGHA2**	Immunoglobulin alpha-2 heavy chain	0.16455	0.22916	0.32699
**SAA2**	Serum amyloid A-2 protein	0.16262	0.54091	0.11322
**C3**	Complement C3	0.15873	0.28752	0.42571
**CD5L**	CD5 antigen-like	0.15522	0.28735	0.24657
**APOH**	Beta-2-glycoprotein 1	0.14845	0.08017	0.11993
**APOC4**	Apolipoprotein C-IV	0.13877	0.34145	0.88349
**IGKV2D-30**	Immunoglobulin kappa variable 2D-30	0.13635	0.25257	0.31321
**IGKV3-20**	Immunoglobulin kappa variable 3-20	0.13230	0.21221	0.26747
**F2**	Prothrombin	0.12753	0.14426	0.17252
**DEFA1**	Neutrophil defensin 1	0.12084	0.06487	0.16673
**IGKV3-11**	Immunoglobulin kappa variable 3D-11	0.11555	0.28258	0.30299
**HBD**	Hemoglobin subunit delta	0.11104	0.21089	0.24000
**IGHV3-15**	Immunoglobulin heavy variable 3-15	0.09907	0.14231	0.14547
**HPX**	Hemopexin	0.09316	0.06820	0.07239
**C4BPA**	C4b-binding protein alpha chain	0.08749	0.14509	0.11371
**CFH**	Complement factor H	0.08478	0.05942	0.05698
**AHSG**	Alpha-2-HS-glycoprotein	0.08214	0.06656	0.06601
**ITIH4**	Inter-alpha-trypsin inhibitor heavy chain H4	0.07791	0.09144	0.08262
**ORM1**	Alpha-1-acid glycoprotein 1	0.06949	0.06656	0.09893
**C4B**	Complement C4-B	0.06570	0.10955	0.14333
**FCN2**	Ficolin-2	0.06421	0.16730	0.14593
**IGKV4-1**	Immunoglobulin kappa variable 4-1	0.05619	0.08108	0.07322
**PLG**	Plasminogen	0.05075	0.15136	0.10656
**IGHV4-34**	Immunoglobulin heavy variable 4-34	0.04754	0.09784	0.08056
**CFHR2**	Complement factor H-related protein 2	0.04753	0.01831	0.01563
**IGHV3-7**	Immunoglobulin heavy variable 3-7	0.04743	0.12237	0.13869

References

(1) Francia, V.; Reker-Smit, C.; Boel, G.; Salvati, A. Limits and challenges in using transport inhibitors to characterize how nano-sized drug carriers enter cells. *Nanomedicine* 2019, 14, 1533-1549.

(2) Rennick, J. J.; Johnston, A. P.; Parton, R. G. Key principles and methods for studying the endocytosis of biological and nanoparticle therapeutics. *Nat. Nanotech.* 2021, 1-11.

(3) Chaudhary, N.; Gomez, G. A.; Howes, M. T.; Lo, H. P.; McMahon, K.-A.; Rae, J. A.; Schieber, N. L.; Hill, M. M.; Gaus, K.; Yap, A. S.; Parton, R. G. Endocytic crosstalk: cavins, caveolins, and caveolae regulate clathrin-independent endocytosis. *PLoS Biol.* 2014, 12 (4), e1001832.

(4) Olivieri, P. H.; Jesus, M. B.; Nader, H. B.; Justo, G. Z.; Sousa, A. A. Cell-surface glycosaminoglycans regulate the cellular uptake of charged polystyrene nanoparticles. *Nanoscale* 2022, 14 (19), 7350-7363.

(5) Regoeczi, E.; Chindemi, P. A.; Hu, W. L. Interaction of transferrin and its iron-binding fragments with heparin. *Biochem. J*. 1994, 299, 819–823.
